# Supplementary material for: Neutrophils Recruited by IL-22 in Peripheral Tissues Function as TRAIL-Dependent Antiviral Effectors against MCMV
Source: Cell Host Microbe. 2014 Apr 9;15(4):471–83. doi: 10.1016/j.chom.2014.03.003 (PMC3989063; doi:10.1016/j.chom.2014.03.003)
Supplement: Document S1. Figures S1–S4, Table S1, and Supplemental Experimental Procedures [file mmc1.pdf]

Cell Host & Microbe, Volume *15*

Supplemental Information

Neutrophils Recruited by IL-22 in Peripheral Tissues Function as TRAIL-Dependent Antiviral Effectors against MCMV

Maria A. Stacey, Morgan Marsden, Tu Anh Pham N, Simon Clare, Garry Dolton, Gabrielle Stack, Emma Jones, Paul Klenerman, Awen M. Gallimore, Philip R. Taylor, Robert J. Snelgrove, Trevor D. Lawley, Gordon Dougan, Chris A. Benedict, Simon A. Jones, Gavin W.G. Wilkinson, and Ian R. Humphreys

Figure S1

Lung

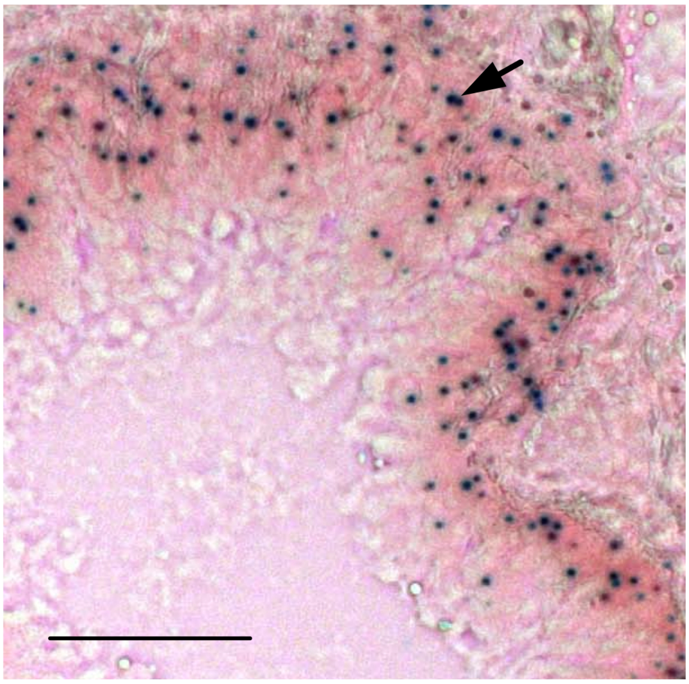

Liver

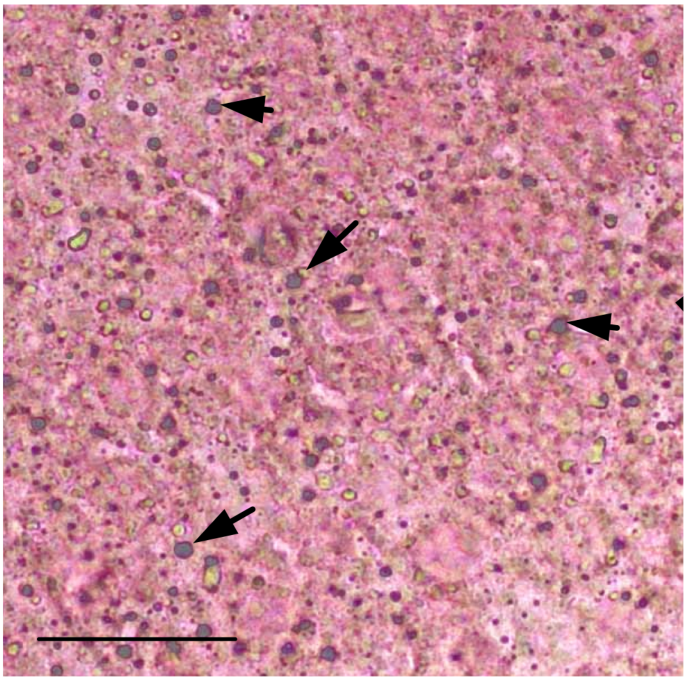

Figure S1, Related to Figure 1: IL-22R is expressed in the lung and liver. To assess *Il22ra1*-driven β-galactosidase reporter activity, lung (top) and liver (bottom) sections from *IL-22ra1*-LacZ reporter mice were incubated in X-gal. Arrows depict $LacZ^+$ epithelial cells (lung) and hepatocytes (liver). Bar = 20μM in all images.

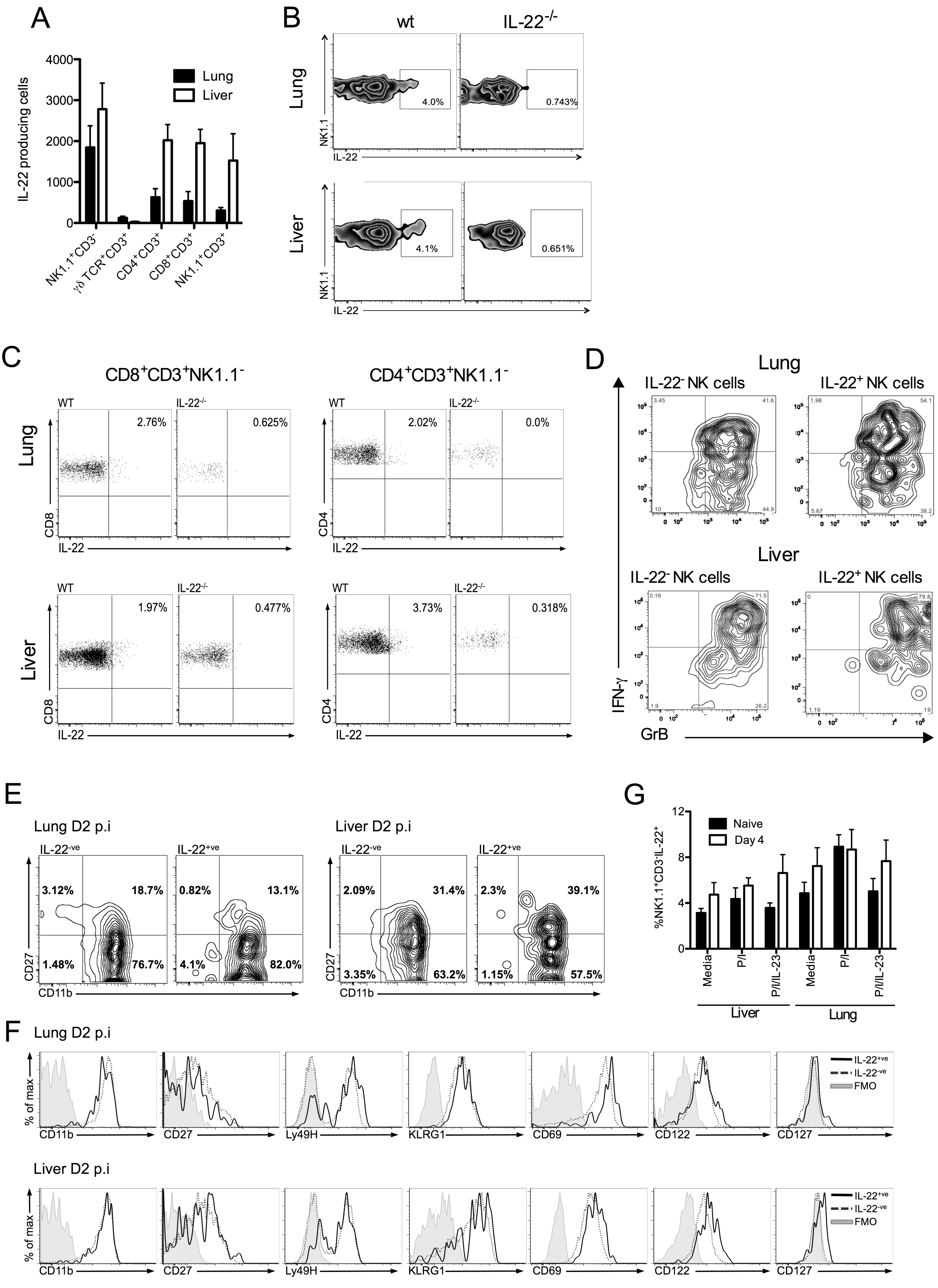
A
B
C
D
E
F
G
Lung
Liver
wt
IL-22-/-
Naive
Day 4
Lung D2 p.i
Liver D2 p.i
IL-22 producing cells
NK1.1
IL-22
IFN-γ
GrB
CD27
CD11b
% of max
IL-22+ NK cells
IL-22- NK cells
CD8+CD3+NK1.1-
CD4+CD3+NK1.1-
%NK1.1+CD3-IL-22+
Media
P/I
P/I/IL-23

Figure S2

Figure S2, Related to Figure 2. IL-22 is expressed by conventional NK cells and T cells in response to MCMV infection. (A) IL-22 expressing leukocytes in lung and liver tissue 2 days post-MCMV infection were quantified by flow cytometry. Results are shown as mean ± SEM of 5 mice. (B) Representative plots of IL-22 expression by $NK1.1^{+}CD3^{-}$ cells in wt and $IL\text{-}22^{-/-}$ mice d2 pi. (C) Representative FACS plots of IL-22 expression by wt and $IL\text{-}22^{-/-}$ $CD8^{+}CD3^{-}NK1.1^{-}$ (left) and $CD4^{+}CD3^{-}NK1.1^{-}$ (right) isolated from the lung (top) and liver (bottom) 4 days post-infection. (D) Representative plots of granzyme B and IFN–γ co-expression by $IL\text{-}22^{+}$ and $IL\text{-}22^{-}$ cells isolated from the lungs and livers 4 days pi. (E) Representative FACS plots of CD27 and CD11b co-expression by $IL\text{-}22^{+}$ and $IL\text{-}22^{-}$ NK cells in the lung and liver d2 pi and (F) representative histogram overlays of expression by $IL\text{-}22^{+}$ (solid line) and $IL\text{-}22^{-}$ (dotted line) of NK cell markers. Results represent 2 experiments. (G) IL-22 expression by $NK1.1^{+}CD3^{-}$ cells from naïve and MCMV-infected mice after 4 hours incubation in the presence of brefeldin A, PMA/ionomycin or PMA/ionomycin/IL-23. Mean ± SEM of 4 mice/group is shown. Data is representative of 2 independent experiments.

Figure S3

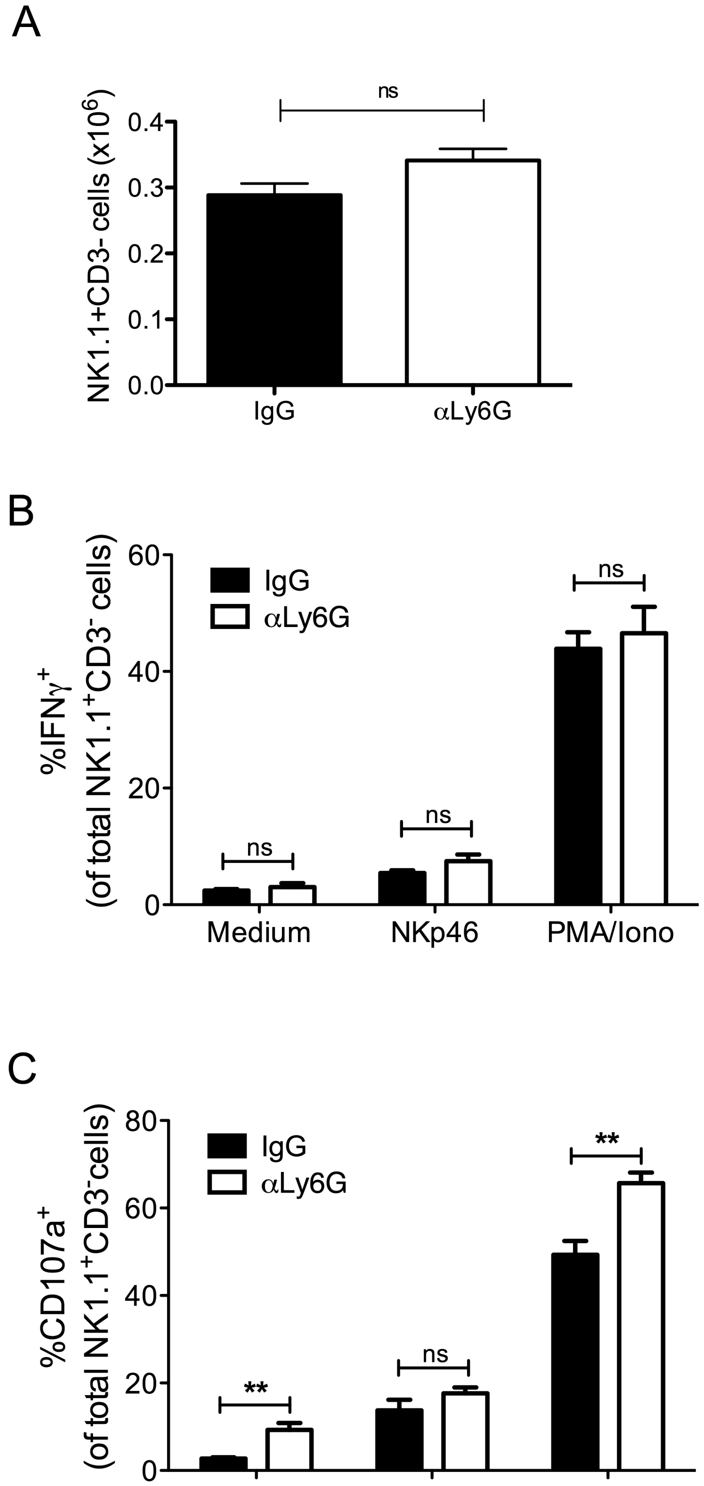

A
ns
NK1.1+CD3- cells (x10^6)
0.0
0.1
0.2
0.3
0.4
IgG
αLy6G
B
IgG
αLy6G
%IFNγ+ (of total NK1.1+CD3- cells)
0
20
40
60
ns
ns
ns
Medium
NKp46
PMA/Iono
C
IgG
αLy6G
%CD107a+ (of total NK1.1+CD3-cells)
0
20
40
60
80
**
ns
**
Medium
NKp46
PMA/Iono

Figure S3, Related to Figure 6. Neutrophil depletion during MCMV infection does not impair NK cell function. MCMV-infected mice were treated with IgG (closed bars) or αLy6G (open bars). (A) After 4 days, leukocytes were isolated from the liver and $NK1.1^{+}CD3^{-}$ cells were quantified. (B & C) CD107a mobilization (B) and IFN–γ (C) expression by $NK1.1^{+}CD3^{-}$ cells was measured following 4 hours of incubation in the presence of monensin (A) or brefeldin A (B) in media +/- plate-bound αNKp46 or PMA/ionomycin. Results are expressed as % positive of all $NK1.1^{+}$ $CD3^{-}$ cells, as assessed by flow cytometry. Mean + SEM in all graphs is shown from 6 mice per group. **p≤0.01, ns=not significant.

Figure S4

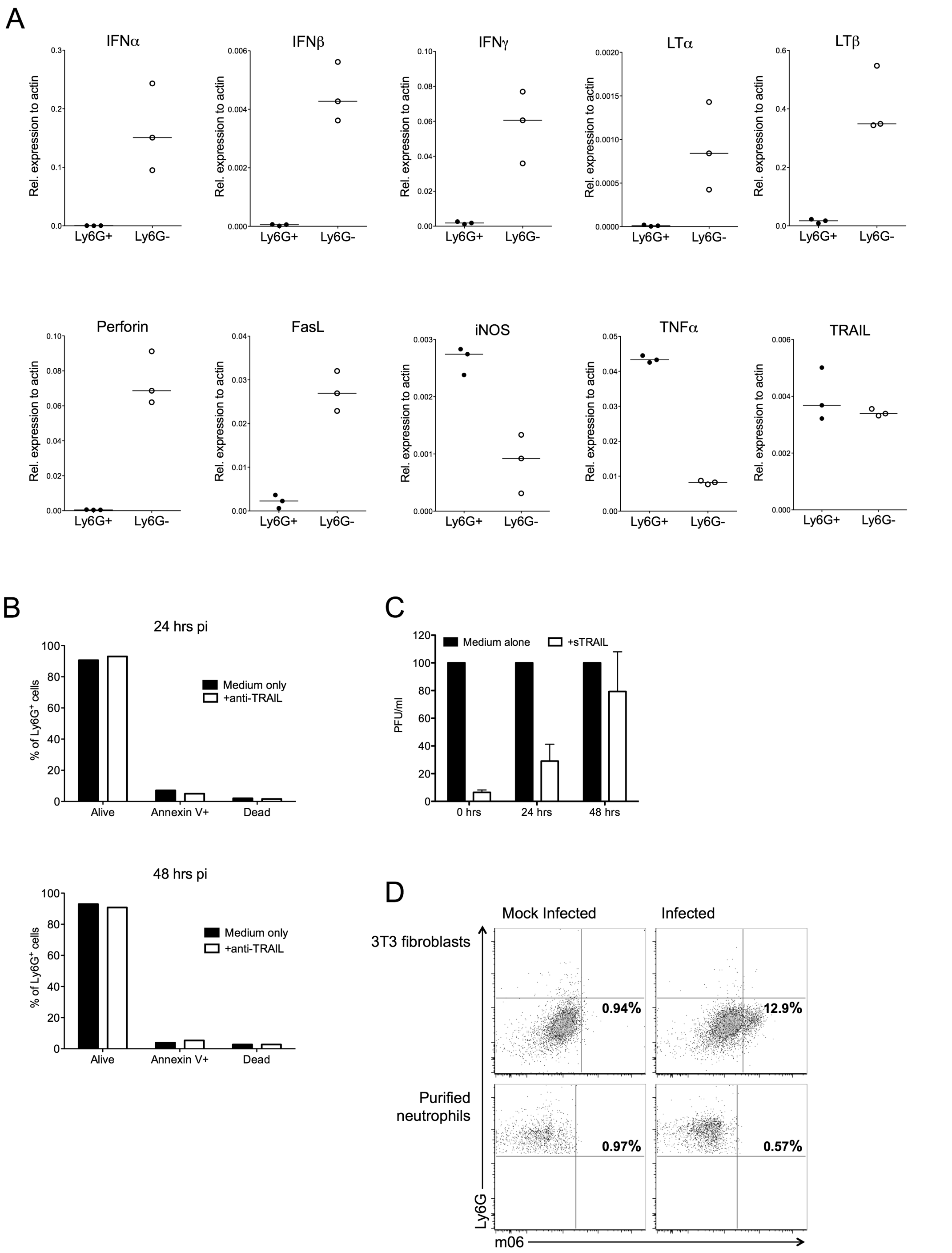
A
IFNα
IFNβ
IFNγ
LTα
LTβ
Perforin
FasL
iNOS
TNFα
TRAIL
Rel. expression to actin
Ly6G+
Ly6G-
B
24 hrs pi
48 hrs pi
% of Ly6G+ cells
Medium only
+anti-TRAIL
Alive
Annexin V+
Dead
C
Medium alone
+sTRAIL
PFU/ml
0 hrs
24 hrs
48 hrs
D
Mock Infected
Infected
3T3 fibroblasts
Purified neutrophils
0.94%
12.9%
0.97%
0.57%
Ly6G
m06

Figure S4, Related to Figure 7. Neutrophils limit MCMV replication via TRAIL and are not susceptible to MCMV infection. (A) Ly6G$^+$ and Ly6G$^-$ cells were purified from livers of MCMV-infected mice 2 days after infection and mRNA expression of antiviral effector genes assessed by qPCR. Individual mice + mean are shown and results represent 2 experiments. (B) Purified Ly6G$^+$ neutrophils were co-incubated with MCMV-infected 3T3 fibroblasts (MOI=0.02), and after 24 (top) and 48 (bottom) hours, neutrophil survival was assessed by the absence of Annexin V and live/dead aqua staining. Data represents 2 separate experiments. (C) Soluble TRAIL was added or not to MCMV-infected fibroblasts 0, 24 or 48 hours post-MCMV infection and virus production in supernatants were measured 7 days later. Data from 3 merged experiments are shown. (D) Purified Ly6G$^+$ neutrophils or 3T3 fibroblasts were mock-infected or infected with MCMV (MOI=0.2) and 24 hours later productively-infected cells were detected with αm06 antibody.

Supplementary Table 1, Related to Experimental Procedures. Primer sequences for qPCR analysis of gene expression.

Gene of interest	Forward primer	Reverse primer
β-actin	GGGCTATGCTCTCCCTCAC	GATGTCACGCACGATTTCC
IFNα	GTCAGTGTCAGAAGCTCCTGTGGC	CTATGGTCCAGGCACAGTGACTG
IFNβ	GAATCTCTCCTTTCTCCTG	CTGACAACCTCCCAGGCAC
IFNγ	GCCGTGGCAGTAACAGCC	AACGCTACACACTGCATCTGGG
LTα	AACCCAAGAATTGGATTCCAGG	TGTGACCCTTGAAACAACGGT
LTβ	TGTCTCCAGCTGCGGATTCTA	TTTGGCAGCTGTTGAACCC
Perforin	GAGAAGACCTATCAGGACCA	AGCCTGTGGTAAGCATG
FasL	TGAATTACCCATGTCCCCAG	AAACTGACCCTGGAGGAGCC
iNOS	GGCAGCCTGTGAGACCTTTG	GCATTGGAAGTGAAGCGTTTC
TNFα	CATCTTCTCAAAATTCGAGTGACAA	TGGGAGTAGACAAGGTACACCCC
TRAIL	CCTCTCGGAAAGGGCATTC	TCCTGCTCGATGACCAGCT
IL-22R	CTACGTGTGCCGAGTGAAGA	AAGCGTAGGGGTTGAAAGGT

Supplemental Experimental Procedures

IL-22R Reporter mice

The IL-22RA1 reporter mice were generated as part of the Sanger Mouse Genetics Project (MGP, www.knockoutmouse.org) using JM8 ES cells on a C57BL/6N background (Pettitt et al., 2009; Skarnes et al., 2011). The targeted *Il22ra1*$^{tm1a(KOMP)Wtsi}$ allele comprised a promoter-driven targeted DNA cassette encoding a neomycin-resistance marker, inserted into the first intron (http://www.sanger.ac.uk/mouseportal/search?query=il22ra1). The activity of the splice acceptor site within the neomycin cassette formed a truncated, non-functional mRNA containing only one of seven exons (ENSMUSE00000661894).

Flow cytometry

Leukocytes were stained for surface and intracellular markers with a combination of the following antibodies (from Biolegend, BD Biosciences, eBioscience, R and D Systems or Serotec): Ly6G (1A8), CD11b (M1/70), Ly6B (7/4), F4/80 (BM8), NK1.1 (PK136), CD3 (145-2C11), TCRγδ (GL3), Gr1 (RB6-8C5), CD4 (RM4-5), CD8 (53-6.7), TRAIL (N2B2), Ly49H (3D10), KLRG-1 (2F1), CD122 (TM-β1), CD27 (LG.3A10), CD127 (A7R34), CD69 (H1.2F3), IL-22 (IC582P) αIFN–γ (XMG1.2) and granzyme B (clone GB11). In some experiments, cells were also stained with αRORγT (clone B2D, eBioscience) according to published methodology (Sanos and Diefenbach, 2010). MCMV infection of neutrophils and 3T3 cells was assessed by intracellular staining with αm06 conjugated to APC (Innova biosciences).

All data were acquired on a BD FACS Canto II. Electronic compensation was performed with antibody-capture beads (BD Biosciences) stained separately with individual antibodies used in the experimental panel. A minimum of 30,000 events was acquired in each case and data analyzed using FlowJo software version 8.5.3 (TreeStar Inc.). Total numbers of different cell populations were calculated by multiplying the total number of viable leukocytes (assessed by trypan blue exclusion) by percent positive cells, as detected by flow cytometry.

Histological analysis of neutrophil and MCMV co-localization

To identify MCMV and neutrophil co-localization, paraffin-embedded liver sections were rehydrated after deparaffinising. Sections were then incubated with pre-warmed 0.01% trypsin for 15mins at 37°C. After blocking steps, sections were stained overnight 4°C with 2μg/ml αm123 (Croma 101, a kind gift from Stipan Jonjić, University of Rijeka) and visualized with DAB (Vector Labs). Slides were then counterstained with 2μg/ml αLy6G (1A8, Bioxcell) for 1 hour at room temperature, visualized with VIP Chromogen solution (Vector Labs) and counterstained with hematoxylin. Images were acquired using a Nikon Eclipse 80I microscope and Nikon ACT1 software.

Assessment of Ly6G^{+} and Ly6G^{-} gene expression

To examine neutrophil-specific gene expression, leukocytes were isolated from the livers of MCMV-infected mice, stained at 4°C with αLy6G-PE and separated by positive selection with αPE beads according to manufacturers

instructions (Miltenyi Biotec). RNA from positive (>80% $Ly6G^+$) and negative fractions (<2% $Ly6G^+$) was then extracted using an RNAeasy plus kit (Qiagen), treated with DNAse and cDNA was synthesized with a TaqMan reverse transcription kit (Applied Biosystems). Tissue RNA was extracted with RNAeasy kit and cDNA was synthesized as above. Gene expression was then assayed quantitative PCR using a Mini Opticon (Bio-Rad) and Platinum SYBR green mastermix reagent (Biorad). All primer sequences for gene detection are provided in Supplemental Table 1.

Neutrophil purification for *in vitro* killing assay

To purify neutrophils from $RAG^{-/-}$ spleens, splenocytes were first incubated with EasySep mouse neutrophil enrichment negative selection cocktail, washed and incubated with EasySep biotin selection cocktail. The cells were then incubated with EasySep magnetic particles and run through an LS column (Miltenyi). The negative fraction was then subjected to αLy6G-PE staining, and positive selection as described above. The purity of the separated cell fractions were assessed by flow cytometry to be ≥97%. In some experiments, neutrophils were isolated on days stated in legends and neutrophil death/apoptosis assessed with Live/Dead Fixable Aqua (Invitrogen) and Annexin-V (BD Biosciences), according to manufacturer's instructions.

Supplemental References

Pettitt, S.J., Liang, Q., Rairdan, X.Y., Moran, J.L., Prosser, H.M., Beier, D.R., Lloyd, K.C., Bradley, A., and Skarnes, W.C. (2009). Agouti C57BL/6N embryonic stem cells for mouse genetic resources. Nat Methods *6*, 493-495.

Sanos, S.L., and Diefenbach, A. (2010). Isolation of NK cells and NK-like cells from the intestinal lamina propria. Methods Mol Biol *612*, 505-517.

Skarnes, W.C., Rosen, B., West, A.P., Koutsourakis, M., Bushell, W., Iyer, V., Mujica, A.O., Thomas, M., Harrow, J., Cox, T., *et al.* (2011). A conditional knockout resource for the genome-wide study of mouse gene function. Nature *474*, 337-342.
