# Supplementary material for: Neutrophils Recruited by IL-22 in Peripheral Tissues Function as TRAIL-Dependent Antiviral Effectors against MCMV
Source: Cell Host Microbe. 2014 Apr 9;15(4):471–83. doi: 10.1016/j.chom.2014.03.003 (PMC3989063; doi:10.1016/j.chom.2014.03.003)
Supplement: Document S2. Article plus Supplemental Information [file mmc2.pdf]

Cell Host & Microbe
Article

Neutrophils Recruited by IL-22 in Peripheral Tissues Function as TRAIL-Dependent Antiviral Effectors against MCMV

Maria A. Stacey,[1] Morgan Marsden,[1] Tu Anh Pham N,[2] Simon Clare,[2] Garry Dolton,[1] Gabrielle Stack,[1] Emma Jones,[1] Paul Klenerman,[3] Awen M. Gallimore,[1] Philip R. Taylor,[1] Robert J. Snelgrove,[4] Trevor D. Lawley,[2] Gordon Dougan,[2] Chris A. Benedict,[5] Simon A. Jones,[1] Gavin W.G. Wilkinson,[1] and Ian R. Humphreys[1,*]
[1]Institute of Infection and Immunity, School of Medicine, Cardiff University, Cardiff CF14 4XN, Wales, UK
[2]Microbial Pathogenesis Laboratory, Wellcome Trust Sanger Institute, Hinxton, Cambridgeshire CB10 1HH, UK
[3]Nuffield Department of Medicine, University of Oxford, Oxford OX1 3SY, UK
[4]Imperial College London, Leukocyte Biology Section, National Heart and Lung Institute, London SW7 2AZ, UK
[5]Division of Immune Regulation, La Jolla Institute for Allergy and Immunology, 9420 Athena Circle, La Jolla, CA 92037, USA
*Correspondence: humphreysir@cardiff.ac.uk
http://dx.doi.org/10.1016/j.chom.2014.03.003
This is an open access article under the CC BY license (http://creativecommons.org/licenses/by/3.0/).

SUMMARY

During primary infection, murine cytomegalovirus (MCMV) spreads systemically, resulting in virus replication and pathology in multiple organs. This disseminated infection is ultimately controlled, but the underlying immune defense mechanisms are unclear. Investigating the role of the cytokine IL-22 in MCMV infection, we discovered an unanticipated function for neutrophils as potent antiviral effector cells that restrict viral replication and associated pathogenesis in peripheral organs. NK-, NKT-, and T cell-secreted IL-22 orchestrated antiviral neutrophil-mediated responses via induction in stromal nonhematopoietic tissue of the neutrophil-recruiting chemokine CXCL1. The antiviral effector properties of infiltrating neutrophils were directly linked to the expression of TNF-related apoptosis-inducing ligand (TRAIL). Our data identify a role for neutrophils in antiviral defense, and establish a functional link between IL-22 and the control of antiviral neutrophil responses that prevents pathogenic herpesvirus infection in peripheral organs.

INTRODUCTION

During acute infection, pathogenic viruses target numerous organs to facilitate replication and dissemination. The mechanisms of organ-specific control of virus infection are poorly understood.

The β-herpesvirus murine cytomegalovirus (MCMV) has coevolved with its mammalian host over millions of years, providing a paradigm of a well-adapted persistent virus that has been extensively exploited in studies of host-pathogen interactions in vivo. MCMV also provides the most tractable in vivo model for the pathogenic β-herpesvirus human cytomegalovirus (HCMV), exhibiting many parallels in terms of pathogenesis, host immunity, immune evasion, and broad tissue tropism (Shellam et al., 2006). NK cells are a key component of the innate immune response and are critical for the control of human herpesviruses, a control that has been elegantly modeled in MCMV (Biron et al., 1989; Bukowski et al., 1984). Importantly, however, the antiviral role of NK cells can be both cell-type and organ specific. For example, NK cell depletion preferentially increases MCMV progeny derived from endothelial cells as compared with nonendothelial cell-derived virus, and this effect is more profound in the lung versus other sites of infection (Sacher et al., 2012). Moreover, NK cells in the salivary gland, which represents a key site of MCMV persistence and dissemination, are hyporesponsive to MCMV infection (Tessmer et al., 2011).

Studies in MCMV also highlight the pivotal role for cytokines such as type I interferons (IFNαβ), lymphotoxin, IL-12, and IL-18 in either inhibiting viral replication directly or regulating the development of innate and adaptive immunity (Andoniou et al., 2005; Andrews et al., 2003; Banks et al., 2005; Orange and Biron, 1996). However, restricted expression of such cytokines in MCMV-infected tissues is observed (Schneider et al., 2008). Collectively, these data are consistent with the existence of additional antiviral effector mechanisms that counter CMV in a broad range of cells within a plethora of tissue microenvironments.

Interleukin-22 (IL-22) is an important effector cytokine in peripheral tissues. IL-22 is expressed by numerous innate and adaptive immune cells and signals through the IL-22Rα/IL-10Rβ dimeric receptor (Sonnenberg et al., 2011). While IL-10Rβ is ubiquitously expressed, IL-22Rα expression is restricted to nonhematapoetic cells, with elevated expression in tissues such as the oral/gastrointestinal tract, lung, skin, kidney, and liver (Wolk et al., 2004).

IL-22 contributes to the immune control of gram-negative bacterial infections at mucosal surfaces while also exhibiting tissue-protective functions (Aujla et al., 2008; Zenewicz et al., 2007; Zheng et al., 2008). The role of IL-22 in viral infections is less well defined. IL-22 neutralization does not impair protection from influenza infection in mice (Guo and Topham, 2010) and, in certain viral infection models, can heighten inflammation without influencing virus clearance (Zhang et al., 2011). In contrast, IL-22 is cytoprotective in the liver during arenavirus chronicity

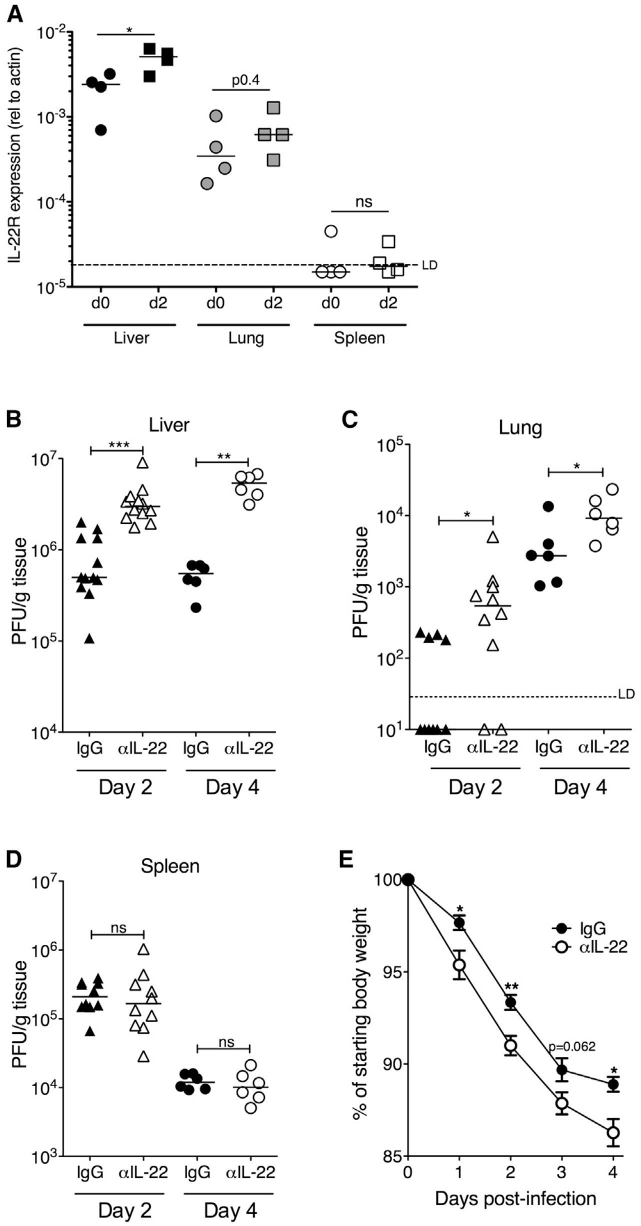

Figure 1. IL-22 Limits Acute MCMV Replication in a Tissue-Restricted Manner

(A) IL-22R gene expression in the liver, lungs, and spleens of naive (day 0) and MCMV-infected mice day 2 p.i.

(B–D) Replicating virus in livers (B), lungs (C), and spleens (D) of mice infected for 2 (triangles) and 4 (circles) days and treated with IgG or αIL-22. Results are expressed as PFU/g tissue. Individual mice + median values are shown. Dotted line, limit of detection (LD).

(E) Weight loss is expressed as mean ± SEM (6 mice/group) of percent of starting weight. Data represent two to six experiments. See also Figure S1.

(Pellegrini et al., 2011). CD161^{+} T cells that express IL-22 are enriched in the liver during chronic hepatitis C virus (HCV) infection (Billerbeck et al., 2010; Kang et al., 2012), and the single nucleotide polymorphism IL-22-rs1012356 SNP is associated with protection from HCV (Hennig et al., 2007). IL-22 has also been implicated in direct inhibition of dengue virus replication (Guabiraba et al., 2013) and T cell-mediated protection from horizontal HIV transmission (Missé et al., 2007). Consequently, a consensus is beginning to emerge that IL-22 may exert antiviral control during infection.

To investigate this, we utilized the MCMV model to elucidate the role that IL-22 plays in viral infection of peripheral tissue. Our results reveal a previously unanticipated mechanism through which IL-22 impacts on virus-induced immune responses and a potent effector mechanism that counters herpesvirus infection.

RESULTS

IL-22 Affords Tissue-Restricted Protection from MCMV Infection

During primary infection, MCMV targets multiple organs of the secondary lymphoid tissue (e.g., spleen), mucosa (e.g., lung), and nonmucosa (e.g., liver). IL-22R mRNA is expressed predominantly in barrier surfaces and also in the liver (Wolk et al., 2004). In accordance, IL-22R was expressed in murine lung and liver, and expression was further elevated in the liver and, to a lesser extent, the lung in response to MCMV (Figure 1A). No significant IL-22R expression was detected in the spleen before or after MCMV infection (Figure 1A). Histological analysis of mice expressing LacZ under the control of the *Il-22ra1* promoter demonstrated IL-22R expression within the lung, particularly by epithelial cells (see Figure S1 available online). Moreover, IL-22R expression by cells with large nuclei, indicative of hepatocytes, was detectable throughout the liver (Figure S1).

Given that IL-22R was expressed in mucosal and hepatic sites targeted by MCMV, we hypothesized that IL-22 influenced the outcome of acute infection. To test this, MCMV-infected mice were treated with either an IL-22-blocking antibody (αIL-22) or IgG control. IL-22 neutralization markedly increased infectious virus load in the liver as early as 2 days after infection (Figure 1B). In the lung, low levels of virus infection occur within the first 2 days of infection, as demonstrated by detection of infectious virus in only four of ten IgG-treated mice (Figure 1C). After αIL-22 treatment, however, virus was detected in eight of ten lungs, seven of which demonstrated higher virus load than IgG-treated mice (Figure 1C), highlighting an antiviral function for IL-22 during initial infection and suggesting it sets a threshold for acute virus replication in the lung. Consistent with low IL-22R expression in the spleen (Figure 1A), IL-22 neutralization did not influence virus replication in this organ (Figure 1D). Despite this tissue-restricted antiviral role for IL-22, the global effect of IL-22 was demonstrated by exacerbated weight loss in αIL-22-treated mice (Figure 1E). Thus, IL-22 limits MCMV infection in an organ-specific manner that is determined by IL-22R expression, and restricts clinical signs of virus-induced disease.

Conventional NK Cells, NK T Cells, and T Cells Are Significant Sources of IL-22 in MCMV Infection

MCMV infection enhanced IL-22 production in the liver and lung (Figure 2A). Conventional NK1.1^{+} NK cells and NK T cells secrete IL-22 in virus infections (Guo and Topham, 2010; Juno et al., 2012; Kumar et al., 2013), and NK1.1 depletion during MCMV infection abrogated IL-22 protein production in the liver and lung (Figure 2A). Furthermore, T cells produce IL-22 in certain

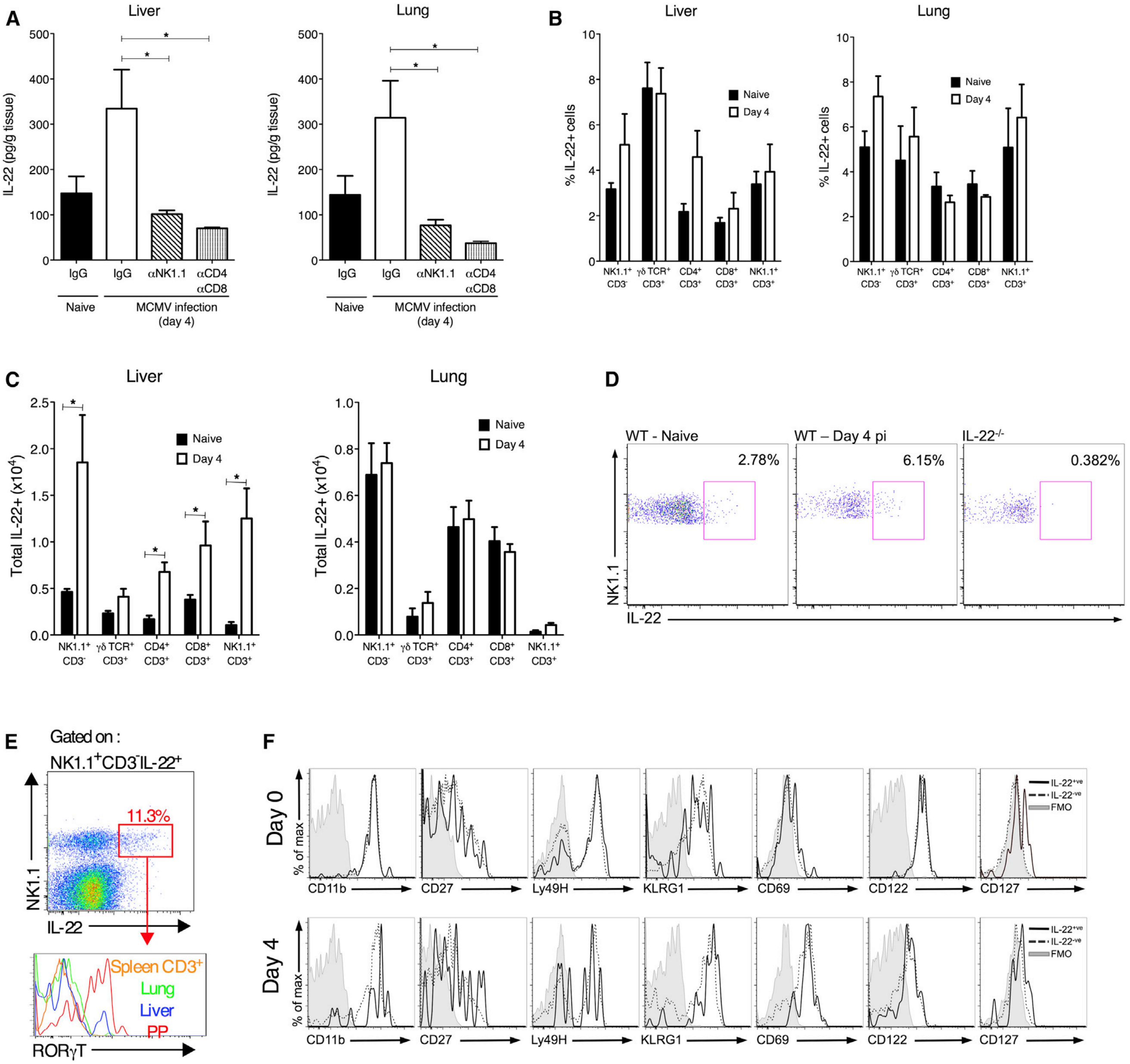

Figure 2. Conventional NK Cells, NK T Cells, and αβ T Cells Express IL-22 during MCMV Infection

(A–C) Mice were infected with MCMV or mock infected (naive), and day 4 p.i. liver (left) and lung (right) tissue was isolated. (A) IL-22 protein concentrations within organ homogenates from naive mice, MCMV-infected mice (day 4 p.i.) treated with IgG, αNK1.1, or αCD4 and αCD8. (B and C) The proportion (B) and total numbers (C) of IL-22$^+$ cells in naive and infected (day 4 p.i.) organs were assessed by FACS. Results show mean ± SEM of four to seven mice/group and represent two (A) or six (B and C) experiments studying expression at either day 2 or day 4 p.i.

(D) Representative bivariate flow cytometry plots of IL-22 versus NK1.1 expression by hepatic NK1.1$^+$CD3$^+$ cells from WT (left and middle) or *IL-22$^{-/-}$* mice day 0 or day 4 p.i., measured after 4 hr stimulation with PMA/ionomycin.

(E) Expression of RORγT by NK1.1$^+$CD3$^-$IL-22$^+$ cells derived from the Peyer's patch (PP), liver and lung, and splenic CD3$^+$ cells from naive mice.

(F) Representative histogram overlays of surface marker expression by IL-22$^+$ (solid line) and IL-22$^-$ (dotted line) pulmonary NK cells day 0 and day 4 p.i. Results represent ≥3 experiments. See also Figure S2.

infections (Sonnenberg et al., 2011), and codepletion of CD4$^+$ and CD8$^+$ cells (which includes CD4$^+$ NK T cells) also reduced IL-22 production (Figure 2A). NK1.1 or CD4/CD8 depletion reduced IL-22 protein concentrations below those detected in naive tissue. This may reflect higher IL-22 turnover upon infection due to increased IL-22R expression (Figure 1A) and/or possible secretion of IL-22 binding protein that, after depletion of IL-22$^+$ cells, reduced detectable soluble IL-22 below levels measured in naive tissue.

In accordance with the hypothesis that NK cells, NK T cells, and CD4$^+$ and CD8$^+$ αβ T cells produce IL-22, analysis of cytokine secretion by leukocytes following ex vivo stimulation with

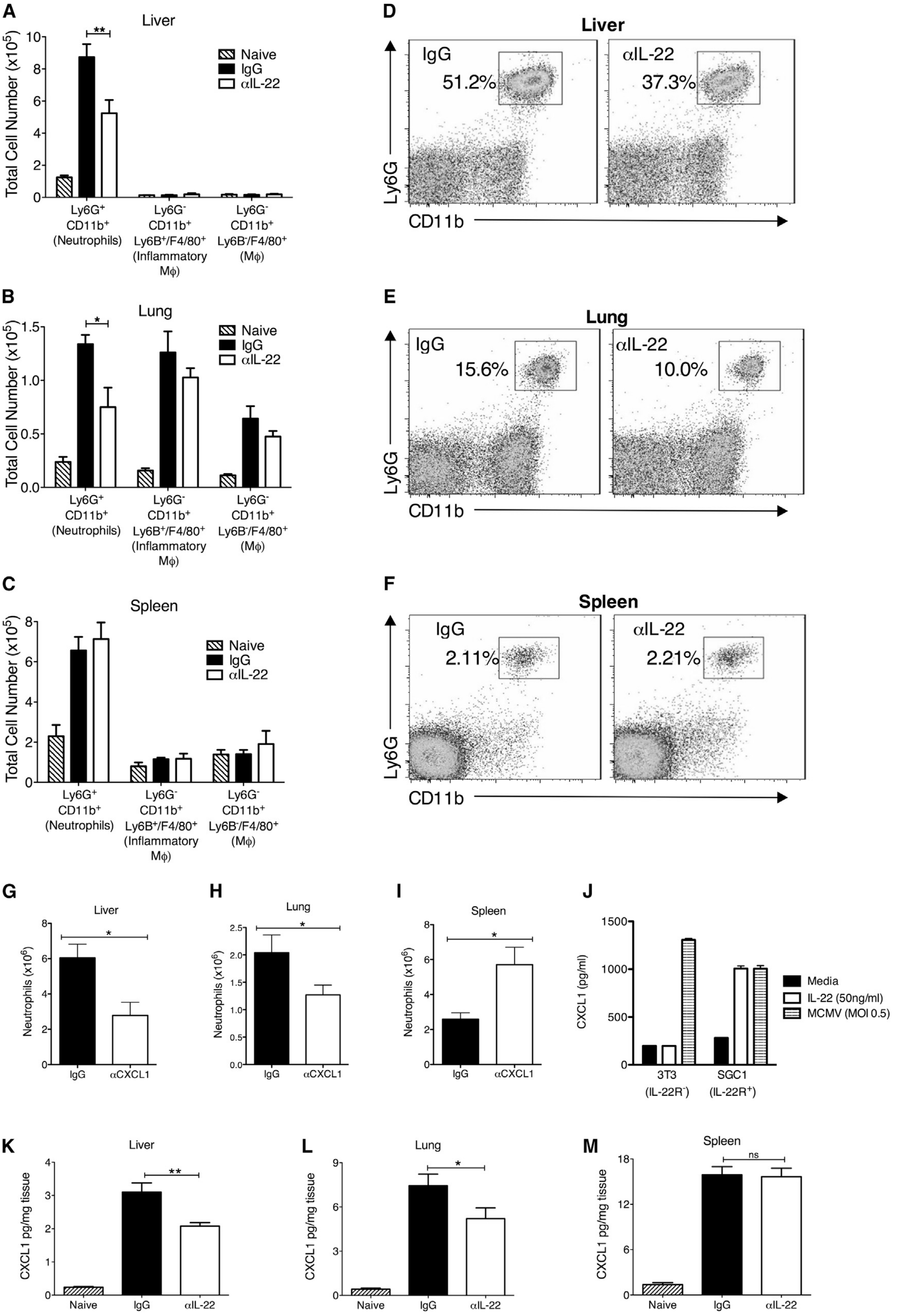

(legend on next page)

PMA/ionomycin and IL-23 identified NK cells (Figures 2B–2E, S2A, and S2B), CD4$^+$CD3$^+$, and CD8$^+$CD3$^+$ (Figures 2B, 2C, S2A, and S2C) to be significant IL-22$^+$ cells in the liver and lung, and NK T cells (NK1.1$^+$CD3$^+$) also representing a significant hepatic IL-22-secreting cell (Figures 2B and 2C). NK cells were the predominant IL-22$^+$ cell-type at day 2 (Figure S2A) and day 4 p.i. (Figures 2B and 2C). Thus, NK1.1$^+$ cells and T cells represent the major IL-22-producing cells in tissues where IL-22 restricts MCMV.

Phenotypic characterization of NK cells demonstrated that, unlike NKp46$^+$CD3$^-$ IL-22 producing innate lymphoid cells (ILCs) of the gastrointestinal tract (Satoh-Takayama et al., 2008), IL-22$^+$ NK cells in the liver and lung did not express RORγT (Figure 2E). Moreover, pulmonary and hepatic IL-22$^+$ NK cells expressed granzyme B, many of which coexpressed IFN-γ, suggesting conventional NK cell function (Figure S2D). Indeed, a significant proportion of IL-22$^+$ NK cells coexpressed CD27 and CD11b (Figures S2E and S2F), indicating high cytotoxic capacity (Hayakawa and Smyth, 2006). Addition of IL-23, the receptor for which is not expressed by conventional NK cells (Cohen et al., 2013), did not increase IL-22$^+$ NK cell frequency detected after incubation with PMA/ionomycin (Figure S2G). Also, IL-22$^+$ NK cells expressed markers of conventional NK cells but not a marker of ILCs, CD127 (Figures 2F, S2E, and S2F). IL-22$^+$ (and IL-22$^-$) NK cells upregulated the activation marker CD69 upon infection (Figures 2F and S2F). Interestingly, IL-22$^+$ and IL-22$^-$ NK cells expressed comparable levels of the activating receptor Ly49H, suggesting the development of IL-22$^+$ NK cells was not influenced by Ly49H activation.

IL-22 Promotes Accumulation of Neutrophils in Peripheral Tissue

The mechanisms through which IL-22 exerted antiviral control were next investigated. Preincubation of an IL-22R-expressing epithelial cell line (SGC1) with IL-22 did not inhibit MCMV replication (data not shown). Moreover, αIL-22 did not influence antiviral IFN (type I and II), lymphotoxin, TNF-α, IL-18, IL-1β, or IL-6 expression, nor did it influence serum amyloid A (SAA) secretion in livers and lungs day 2 p.i. (data not shown); the time point at which IL-22 first displayed antiviral activity. NK cell accumulation and ex vivo degranulation and IFN-γ expression in the lung and liver were unaltered after αIL-22 treatment (data not shown). Moreover, macrophage and monocyte accumulation within MCMV-infected organs was unaffected by αIL-22 treatment (Figures 3A–3C).

Strikingly, however, accumulation of neutrophils, which represented a substantial proportion of the MCMV-elicited leukocyte infiltrate in the liver (Figure 3D) and lung (Figure 3E) 2 days p.i., was substantially reduced in both organs after IL-22 neutralization (Figures 3A, 3B, 3D, and 3E). In the spleen, however, αIL-22 did not influence neutrophil accumulation in this IL-22-refractory site (Figures 3C and 3F).

Murine CXCL1 (KC) is the main neutrophil-activating chemokine (Sadik et al., 2011), and in vivo administration of an αCXCL1 neutralizing antibody restricted neutrophil accumulation in the liver (Figure 3G) and lung (Figure 3H). In contrast, αCXCL1 increased neutrophil accumulation in the spleen (Figure 3I), consistent with the hypothesis that neutrophil migration into the lung and liver is controlled by different mechanisms to those in the spleen. IL-22 induced secretion of CXCL1 by IL-22R$^+$ SGC1 cells by 6 hr poststimulation (Figure 3J), and crucially, αIL-22 reduced CXCL1 protein concentrations in the liver (Figure 3K) and lung (Figure 3L) 2 days p.i., suggesting that early induction of CXCL1 secretion by IL-22 is critical for chemokine-driven neutrophil recruitment. Consistent with the low IL-22R expression in the spleen (Figure 1A), αIL-22 did not influence CXCL1 secretion in this organ (Figure 3M).

Neutrophils Afford Protection from MCMV Replication In Vivo

The association between the tissue-restricted antiviral activity of IL-22 and neutrophil infiltration implied an antiviral function for this cell population. Upon MCMV infection, neutrophils were detected in high numbers in infected tissues (Figures 3A–3C), and a high frequency of activated CD11b$^+$ cells was present in these infected organs (Figure 4A), demonstrating that these cells were activated during MCMV infection. Furthermore, histological analysis of MCMV-infected tissue demonstrated that neutrophils localized adjacent to infected cells (Figure 4B).

To test whether neutrophils contributed to antiviral immunity, MCMV-infected mice were administered with a Ly6G-specific monoclonal antibody that specifically depleted neutrophils without affecting inflammatory monocyte/macrophage populations (Figure 5A). Strikingly, antibody-mediated depletion of neutrophils increased MCMV-induced weight loss (Figure 5B). This exacerbation of virus-induced illness was associated with elevated virus load in liver (Figure 5C) and lungs (Figure 5D), but not the spleen (Figure 5E).

C57BL/6 mice lacking adaptive immunity control acute MCMV infection, a process mediated in part by Ly49H-expressing NK cells recognizing MCMV m157 protein (French et al., 2004). Strikingly, we observed that depletion of neutrophils in MCMV-infected *RAG*$^{-/-}$ mice triggered dramatic weight loss (Figure 5F) and a concurrent large elevation in replicating virus in the livers (Figure 5G) and lungs (Figure 5H). Interestingly, in this model where $\sim$14% of splenocytes prior to MCMV infection are Ly6G$^+$ neutrophils, depletion of these cells also increased replicating virus detected in the spleen (Figure 5I). Thus, neutrophils

Figure 3. IL-22 Neutralization Impairs Neutrophil Recruitment into MCMV-Infected Tissues

MCMV-infected mice were administered IgG or αIL-22 and leukocyte infiltrates (A–F) and chemokine protein expression (K–M) assessed after 2 days. Myeloid cell numbers in liver (A), lung (B), and spleen (C) were quantified and are shown as mean ± SEM of six mice per group. Representative bivariate plots of Ly6G$^+$CD11b$^+$ neutrophils in the liver (D), lung (E), and spleen (F). Results represent three experiments. (G–I) MCMV-infected mice were treated with IgG or αCXCL1 and neutrophil (Ly6G$^+$CD11b$^+$) recruitment into the liver (G), lung (H), and spleen (I) assessed by FACS. Results are expressed as mean + SEM of 11 mice, with data from two independent experiments combined. (J) 3T3 (IL-22R$^-$) and SGC1 (IL-22R$^+$) cells were incubated for 6 hr in medium alone, 50 ng/ml rIL-22, or medium alone prior to MCMV infection (moi 0.5). Supernatants were then assayed for CXCL1 protein. Data are shown as mean + SEM of duplicate samples, representing four experiments. (K–M) CXCL1 protein in liver (K), lung (L), and spleen (M) homogenates from IgG- or αIL-22-treated mice day 2 p.i. Mean + SEM of eight (naive) or ten (MCMV infected ± αIL-22) mice is shown. Results are combined from two independent experiments, representing four in total.

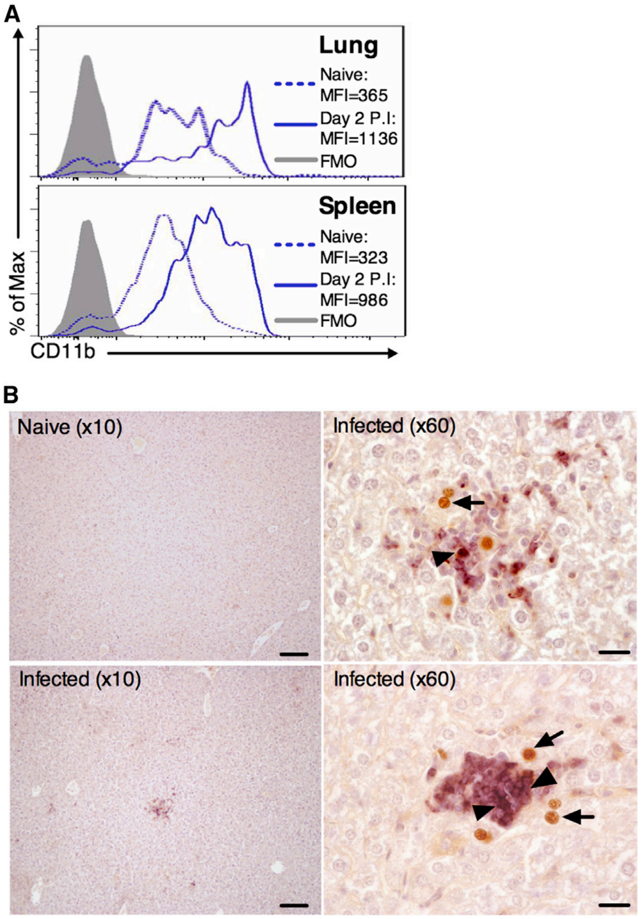

Figure 4. Neutrophil Recruitment into MCMV-Infected Organs

(A) CD11b expression by pulmonary (top) and splenic (bottom) neutrophils in infected (day 2 p.i.) or mock-infected mice.

(B) Neutrophil localization near MCMV-infected (m123$^+$) cells in the liver was visualized in hematoxylin-stained paraffin-embedded sections. Liver sections from mock-infected tissues (10×, top left) were used as negative controls for αm123 (brown) and αLy6G (purple) staining. Infected tissue is shown at 10× magnification (bottom left) and 60× (top and bottom right). All images are taken from different mice, representing <8. Arrow, MCMV-infected m123$^+$ nucleus; triangle, neutrophil. Bars, 100 μM (10× magnification), 20 μM (60× magnification).

are critical early antiviral effector cells during CMV infection in vivo.

Neutrophils Directly Inhibit MCMV Replication

We characterized the mechanistic control of MCMV by neutrophils. Neutrophil crosstalk with NK cells promotes NK cell development, including enhanced NK cell cytotoxicity (Jaeger et al., 2012). However, as mentioned earlier, the accumulation of cytotoxic NK cells was not affected by αIL-22 (data not shown), and neutrophil depletion did not impair NK cell accumulation, cytotoxicity or IFN-γ expression (Figure S3). Furthermore, concurrent depletion of both neutrophils and NK cells exhibited an additive antagonistic effect on host control of infection (Figure 6A). Of note, NK depletion did not influence neutrophil accumulation in infected tissues (data not shown). Taken with the observations that T cells secrete IL-22 (Figures 2A–2C) and that MCMV replication (which is elevated after NK1.1 depletion in this model [Stacey et al., 2011]) directly induces CXCL1 production (Figure 3J), these results demonstrate that NK1.1$^-$ cells are capable of promoting neutrophil recruitment in the absence of NK cells. Importantly, these results also imply that neutrophil activation of NK cells is not the primary mechanism through which neutrophils control MCMV.

We investigated whether neutrophils were capable of direct antiviral activity. Coincubation of purified neutrophils (Figure 6B) with 3T3 fibroblasts led to ~50% neutrophil viability for up to 72 hr and, interestingly, we observed a trend in elevated neutrophil viability following coincubation with MCMV-infected cells (Figure 6C). Crucially, neutrophil coincubation with MCMV-infected fibroblasts dramatically reduced the production of replicative virions in a cell-number-dependent manner (Figure 6D), demonstrating that neutrophils directly inhibit MCMV replication.

Neutrophils Exert Anti-MCMV Activity in a TRAIL-Dependent Manner

To identify the mechanism(s) through which neutrophils exert antiviral activity, expression of putative antiviral effector molecules by hepatic neutrophils isolated 2 days p.i. was measured. We detected no expression of type I and II interferons, perforin, and lymphotoxin by MCMV-induced neutrophils (Figure S4A). However, MCMV-induced neutrophils expressed significant iNOS, TNF-α, TRAIL, and low but reproducibly detectable FasL mRNA (Figure S4A). Gene expression analysis in whole tissue revealed that all four genes were upregulated upon MCMV infection in vivo (Figure 7A).

The effect of antagonizing each of these molecules on antiviral neutrophil activity was next assessed. Strikingly, inhibition of the death receptor ligand TRAIL dramatically abrogated the antiviral activity of neutrophils (Figure 7B) without influencing neutrophil survival (Figure S4B). In addition, FasL blockade moderately antagonized neutrophil-mediated control of MCMV, although this was not statistically significant (Figure 7B). Further, soluble TRAIL (Figure 7C) and, to a lesser extent, soluble FasL (Figure 7D) inhibited MCMV replication, further implying a dominant role for TRAIL in facilitating neutrophil-mediated antiviral control. Indeed, TRAILR (DR5) was upregulated by MCMV-infected fibroblasts in vitro 6 hr p.i., although expression was downregulated by 24 hr with complete reversal of TRAILR expression by 48 hr (Figure 7E). In contrast, we observed no significant regulation of Fas by MCMV (data not shown). Collectively, these results pointed toward a dominant role for TRAIL in the anti-MCMV function of neutrophils and are consistent with the observation that CMVs actively downregulate surface TRAILR expression (Smith et al., 2013). Furthermore, in accordance with complete downregulation of TRAILR expression by 48 hr, delaying the addition of neutrophils (Figure 7F) or soluble TRAIL (Figure S4C) to MCMV-infected fibroblasts until this time point abrogated antiviral activity.

We next investigated whether TRAIL expression by neutrophils contributed to antiviral control in vivo. In accordance with infection-induced TRAIL mRNA expression (Figure 7A), MCMV induced cell-surface TRAIL expression by neutrophils (Figure 7G). We inhibited TRAIL/TRAILR interactions in vivo with blocking αTRAIL antibody in NK-depleted *RAG*$^{-/-}$ mice, thus

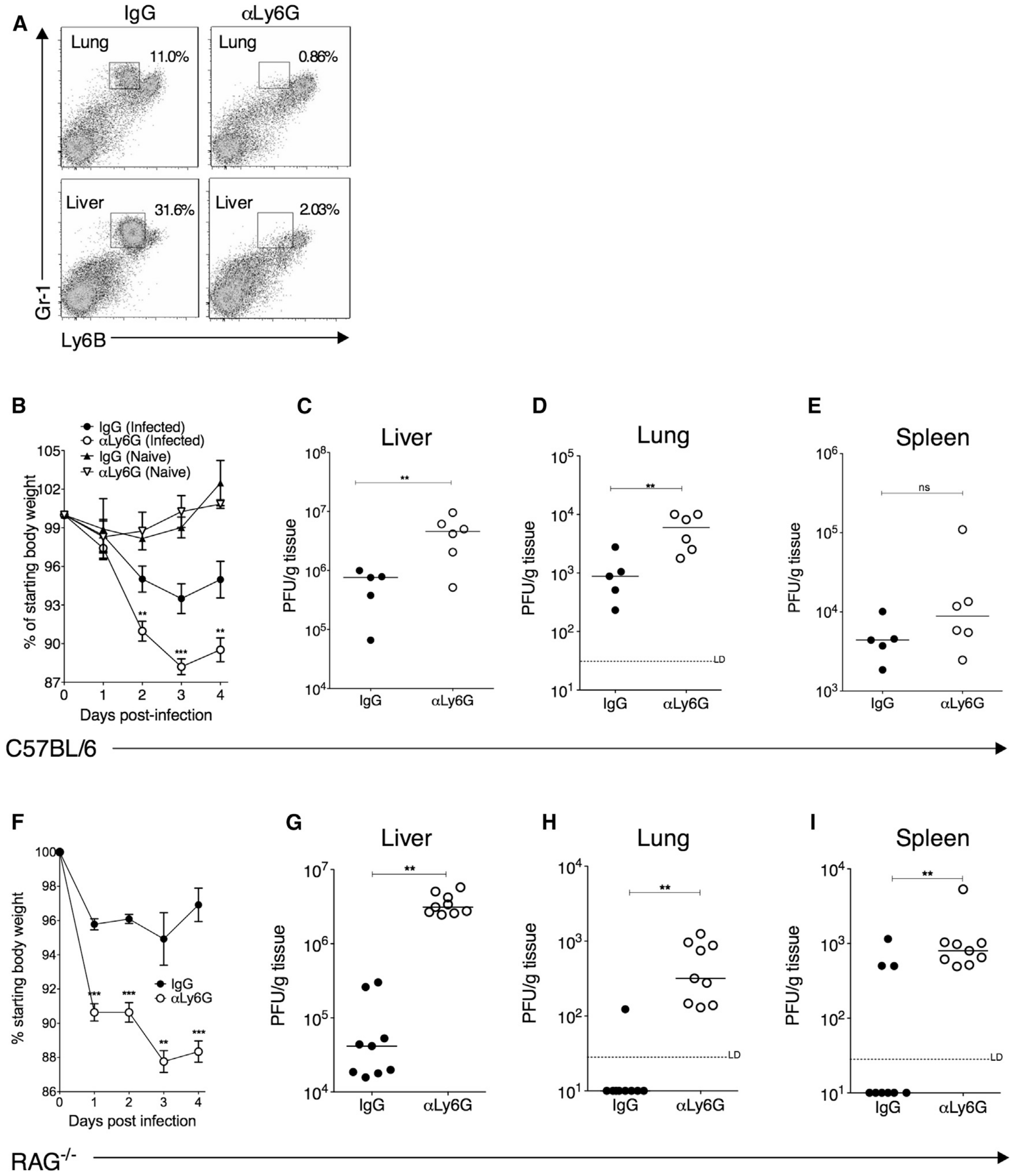

Figure 5. Neutrophil Depletion Impairs Control of Acute MCMV Infection

MCMV-infected and mock-infected C57BL/6 (A–E) or *RAG1*$^{-/-}$ (F–I) mice were treated with αLy6G or IgG. (A) Representative bivariate plots demonstrating specific depletion of neutrophils (Ly6B^{int}Gr1^{hi}) in lungs and livers with αLy6G antibody. (B) Weight loss in MCMV-infected and mock-infected IgG- and αLy6G-treated mice is expressed as percentage of original weight and is shown as mean ± SEM of ten mice per infected group and three mice per naive group. Replicating virus in livers (C), lungs (D), and spleens (E) 4 days p.i. in IgG- and αLy6G-treated mice is shown as individual mice + median. All data represent five independent experiments. (F–I) MCMV-infected *RAG1*$^{-/-}$ mice were treated with IgG or αLy6G. (F) Weight loss is expressed as percentage of original weight and is shown as mean ± SEM of five mice per group, representing two experiments. (G–I) Replicating virus in homogenates from the livers (G), lungs (H), and spleens (I) 4 days p.i. in IgG- and anti-Ly6G-treated *RAG*$^{-/-}$ mice. Individual mice + median from two experiments are shown.

lacking both TRAIL-expressing NK cells and elevated NK cell responsiveness observed in *TRAILR*$^{-/-}$ mice (Diehl et al., 2004) and in αTRAIL-treated *RAG*$^{-/-}$ mice (data not shown). Although NK cell depletion impairs leukocyte recruitment in livers of *RAG*$^{-/-}$ mice (Salazar-Mather et al., 1998), Ly6G depletion experiments demonstrated that neutrophils clearly exerted antiviral control (Figure 7H). Importantly, TRAIL blockade increased MCMV burden to comparable levels measured in neutrophil-depleted mice (Figure 7H). Critically, antagonizing both TRAIL and neutrophils did not further abrogate antiviral protection

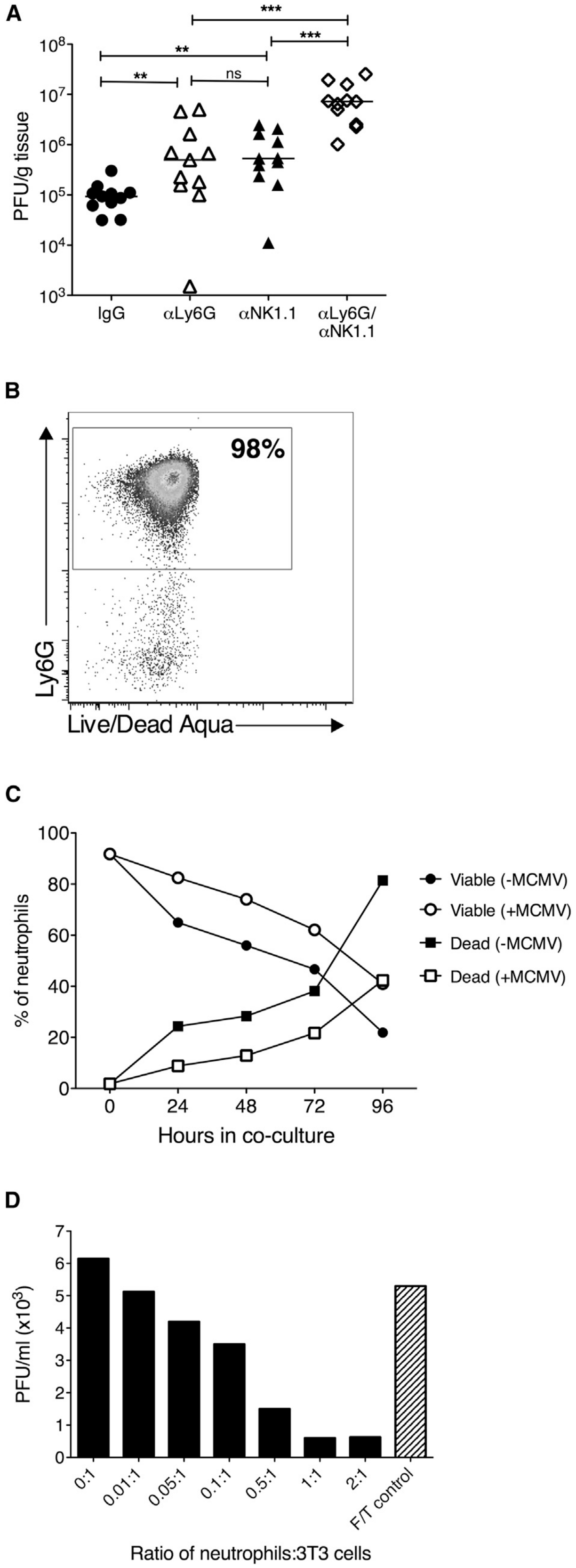

Figure 6. Neutrophils Limit MCMV Replication In Vitro

(A) MCMV-infected mice were treated ± αLy6G ± αNK1.1, and day 4 p.i. virus load was measured by plaque assay. Data from two experiments + median are shown.

(B–D) Neutrophil purity was assessed by FACS (B), and survival following incubation with mock-infected and MCMV-infected fibroblasts was assessed with Annexin V and live/dead aqua (C). (D) Replicating virus in supernatants from neutrophil/fibroblast cocultures was measured after 7 days. Some wells received freeze-thawed neutrophils as negative controls. Data represent three (A), two (C), and four (B and D) experiments. See also Figure S3.

(Figure 7H), providing evidence that neutrophils limit MCMV replication in vivo via TRAIL. Finally, neutralization of IL-22 in neutrophil-depleted WT mice failed to further increase virus load as compared to αLy6G treatment alone, suggesting that IL-22 exerts antiviral control via neutrophil recruitment (Figure 7I). Consistent with this hypothesis, IL-22 neutralization, which resulted in an ~2-fold reduction in neutrophil recruitment, had a lesser impact on antiviral control than Ly6G depletion (Figure 7I). Collectively, these results demonstrate that IL-22 drives antiviral neutrophil response in vivo, and that TRAIL is a significant mechanism through which neutrophils restrict MCMV replication.

DISCUSSION

We identified neutrophils as potent antiviral effector cells that restrict CMV infection of peripheral tissue and exert antiviral activity via TRAIL. In addition, we identified IL-22 as an important component of the antiviral immune response that recruits neutrophils to peripheral sites of MCMV infection in which IL-22R is expressed. Thus, the IL-22-neutrophil axis represents a pathway that counteracts virus invasion of peripheral tissues.

The role that neutrophils play in viral infections is not well appreciated. Depletion of Ly6G$^+$ cells during influenza infection revealed that neutrophils limit influenza-induced illness, although it is not clear whether neutrophils directly impinge on virus replication (Tate et al., 2009, 2011). Paradoxically, neutrophils are associated with influenza-induced inflammation (La Gruta et al., 2007), particularly during infection with highly pathogenic strains (Perrone et al., 2008). In contrast to MCMV, neutrophils support West Nile virus replication, while depletion experiments suggested that neutrophils exert antiviral control during later stages of acute infection (Bai et al., 2010). Moreover, neutrophil extracellular traps capture HIV and promote virus killing via myeloperoxidase and α-defensin (Saitoh et al., 2012) and limit poxvirus infection in vivo (Jenne et al., 2013). Neutrophils also kill HIV-infected CD4$^+$ T cells via antibody-dependent cellular cytotoxicity (Smalls-Mantey et al., 2013). Intriguingly, in the context of herpesviruses, neutropenia has been identified as a risk factor for occurrence of herpesvirus infections in immune-suppressed individuals (Jang et al., 2012; Lee et al., 2012). Importantly, depletion in mice of cells expressing Gr1, which is expressed by numerous cells including Ly6G$^+$ neutrophils (Tate et al., 2009), elevates HSV replication in the genital mucosa (Milligan, 1999) and cornea (Tumpey et al., 1996). We now provide definitive evidence that neutrophils directly inhibit replication of a pathogenic herpesvirus in vitro and in vivo.

Expression of TRAIL is one mechanism through which neutrophils exert direct antiviral activity in vitro and in vivo. Neutrophils

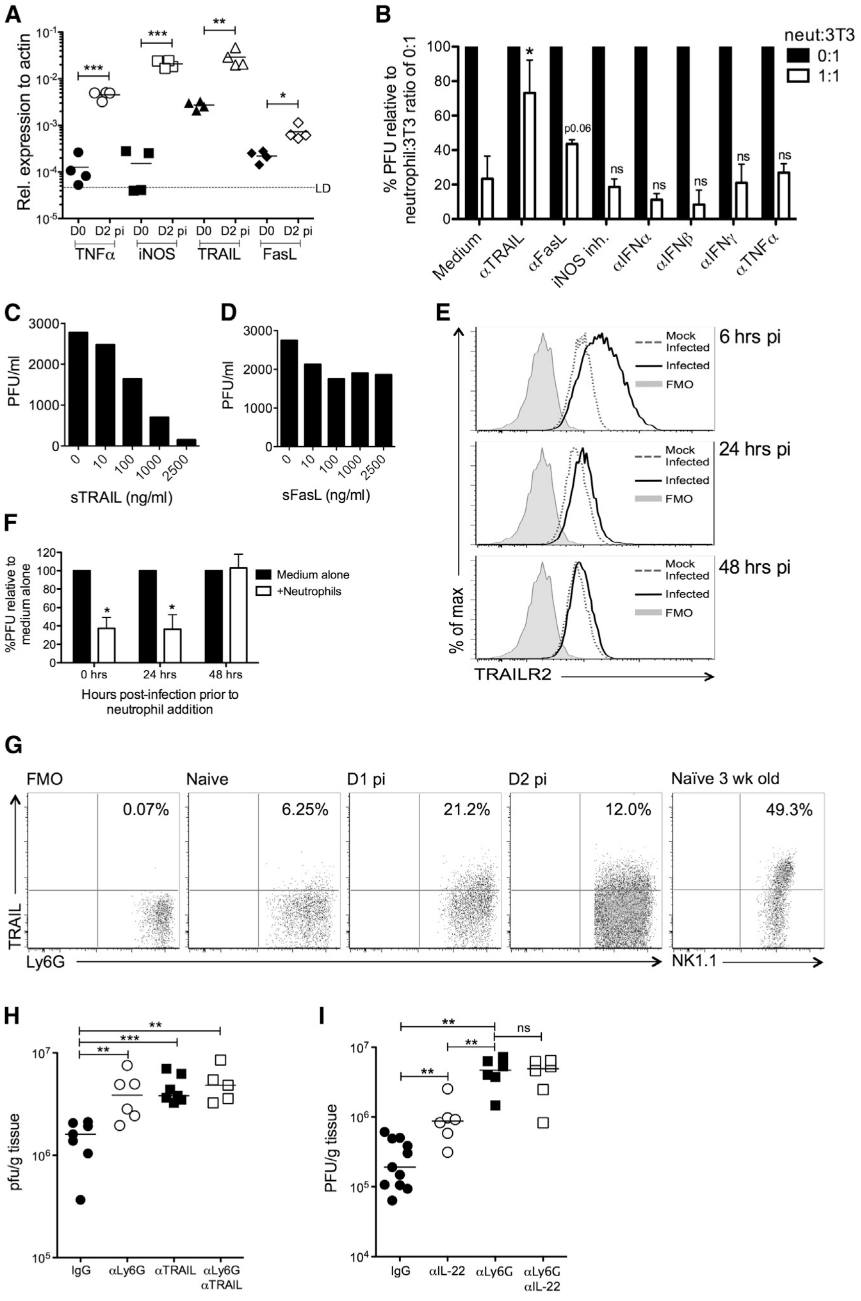

Figure 7. Neutrophils Limit MCMV Replication in a TRAIL-Dependent Manner

(A) Whole-tissue gene expression in naive and infected (day 2 p.i.) livers. (B) Purified neutrophils were added or not at a ratio of 1:1 neutrophils:infected fibroblast ± antagonists of potential antiviral effector molecules. Relative reduction in MCMV PFU was compared between medium control and experimental groups. (C and D) Infected fibroblasts were incubated with recombinant TRAIL (C) or recombinant FasL (D) and replicating virus measured after 7 days. (E) Fibroblasts were MCMV infected (moi 0.2), and TRAILR expression was measured by FACS. Shaded, FMO; dotted lines, mock infected; solid line, MCMV infected. (F) Purified neutrophils were added or not to MCMV-infected fibroblasts 0, 24, or 48 hr p.i. and virus measured 7 days later. Data are shown as mean + SEM of four replicates. (G) Representative bivariant FACS plots of surface TRAIL expression by liver neutrophils. FMO, fluorescence minus one; positive control, $NK1.1^{+}CD3^{-}$ cells from naive livers of 3-week-old mice. (H) MCMV-infected $RAG^{-/-}$ mice were depleted of NK1.1 cells and administered IgG (●), αLy6G (○), αTRAIL (■) or αLy6G and αTRAIL (□), and hepatic virus load was measured at day 4 p.i. (I) MCMV-infected C57BL/6 mice were administered IgG (●), αIL-22 (○), αLy6G (■), or αIL-22 and αLy6G (□) and virus load in liver tissue measured 4 days p.i. Results represent two (A, C, D, F, and G) or three (B and E) independent experiments, or show data merged from two experiments (H and I). See also Figure S4.

expressed TRAIL on the cell surface, although our data do not preclude the possibility that neutrophils also secrete TRAIL in response to MCMV. HCMV UL141 protein promotes intracellular retention of TRAIL receptors, desensitizing cells to TRAIL-mediated apoptosis (Smith et al., 2013). We observed TRAILR downregulation in MCMV-infected fibroblasts, consistent with the role of MCMV m166 protein in restricting TRAILR expression (C.A.B, unpublished data). Abrogation of neutrophil- and TRAIL-mediated inhibition of MCMV replication after 24 hr suggests neutrophils limit virus replication within the first hours of infection when TRAILR is present on the cell surface. TRAILR downregulation implies viral evasion of TRAIL-induced cell death. However, TRAILR signaling also induces NF-κB-mediated proinflammatory cytokine production (Tang et al., 2009), suggesting that TRAIL may induce multiple antiviral pathways. Incomplete reversal of neutrophil antiviral activity following TRAIL inhibition suggests additional mechanisms exist through which neutrophils restrict MCMV. Neutrophil exposure to MCMV or infected cells did not upregulate reactive oxygen species (data not shown), and iNOS, IFNs, iNOS, and TNF-α did not participate singularly in neutrophil antiviral activity. Instead, inhibition of FasL moderately inhibited antiviral function, suggesting neutrophils restrict MCMV via expression of TNFSF proteins.

Neutrophils receive activation signals during migration into inflamed tissue (Futosi et al., 2013), suggesting their activation in MCMV infection may be independent of virus recognition. Indeed, TRAIL expression by neutrophils is rapidly induced by IFNs (Koga et al., 2004; Tecchio et al., 2004). Although neutralization of individual IFNs did not abrogate anti-MCMV neutrophil activity in vitro, it is possible that multiple antiviral cytokines induce antiviral neutrophil activity in vivo without the requirement for pattern recognition receptor-mediated neutrophil activation.

IL-22R expression determined the tissue-restricted influence of IL-22 on antiviral immunity. However, neutrophil depletion in

WT mice also uncovered an organ-restricted antiviral role for neutrophils, with no obvious role for neutrophils in splenic MCMV infection. CXCL1 did not influence neutrophil accumulation within the spleen. Differences in orchestration of splenic versus pulmonary and hepatic neutrophil responses may influence their ability to counter MCMV. MCMV cell tropism within different organs may also influence neutrophil antiviral control. Stromal cells are targeted by MCMV in the spleen (Hsu et al., 2009; Schneider et al., 2008; Verma et al., 2013), and responsiveness of these cells to effector molecules produced by neutrophils may differ from infected cells in the liver and lung. Moreover, the efficiency of viral evasion mechanisms may vary in different cell types, influencing the efficacy of neutrophil antiviral control. Importantly, however, neutrophil depletion in *RAG*$^{-/-}$ mice elevated splenic virus load, demonstrating that neutrophils can limit MCMV infection in the spleen when present in large numbers.

Given the antiviral role of neutrophils, it is surprising that HCMV encodes a homolog of the neutrophil-attractant chemokine CXCL1 (UL146), hypothesized to promote neutrophil recruitment to facilitate HCMV dissemination (Penfold et al., 1999). Host survival in acute infection is essential for establishment of virus persistence and dissemination. Thus, neutrophil limitation of acute infection facilitated by UL146 may be a necessary evil to enable virus chronicity and transmission. Alternatively, UL146 may act preferentially in certain contexts, for example by promoting neutrophil adherence to virus-infected vascular endothelium, but exert less influence within peripheral organs where, as demonstrated in MCMV infection, mammalian CXCL1 is expressed at biologically significant levels. Importantly, MCMV did not productively replicate within neutrophils (Figure S4D). Moreover, we detected very few neutrophils in the salivary glands at the onset of virus persistence (data not shown), supporting the hypothesis that monocytes rather than neutrophils disseminate MCMV (Stoddart et al., 1994). Given that CXCL1 promotes recruitment of nonneutrophils (Tsai et al., 2002), our data imply that UL146 may have a function distinct from neutrophil recruitment.

Of note, MCMV-infected *IL-22R*$^{-/-}$ mice did not exhibit heightened virus load, reduced neutrophil recruitment, or CXCL1 protein in peripheral sites of infection (data not shown). Numerous cytokines can induce proinflammatory chemokines, and heightened proinflammatory cytokine and chemokine responses have been reported following viral infection of *IL-22*$^{-/-}$ mice (Guabiraba et al., 2013), suggesting that alternate proinflammatory/antiviral mechanisms compensate for the absence of the IL-22R signaling in knockout mice, whereas such compensatory mechanisms are absent after IL-22 neutralization in WT mice.

Although NK1.1 depletion revealed that NK and NK T cells secrete virus-induced IL-22, concurrent depletion of NK cells and neutrophils had an additive effect on elevating virus load. Moreover, we observed no decrease in neutrophil recruitment after NK cell depletion (data not shown). Although IL-22-producing T cells in part compensate for the absence of IL-22^{+}NK1.1^{+} cells, MCMV itself is a potent inducer of CXCL1 secretion. This suggests that high virus load after NK cell depletion not only induces significant pathology (van Dommelen et al., 2006) but also is sufficient to promote neutrophil recruitment. Thus, through interactions with the stromal compartment, NK cells may promote *controlled* neutrophil recruitment by restricting virus replication *and* inducing IL-22-dependent neutrophil-activating chemokines.

IL-22 neutralization in neutrophil-depleted mice failed to further increase virus load, suggesting a dominant role of neutrophil recruitment in the antiviral activity of IL-22. However, our study does not preclude the existence of additional antiviral pathways elicited by IL-22R signaling. IL-22 exerts a vast range of biological activities (Ouyang et al., 2011; Sonnenberg et al., 2011; Zenewicz et al., 2007). Indeed, during HIV and influenza infections, IL-22 limits epithelial cell damage at mucosal surfaces (Kim et al., 2012; Kumar et al., 2013). Given the critical role of mucosal surfaces in herpesvirus dissemination and pathogenesis, the role that IL-22 may play in acute and chronic infection of the mucosa warrants further investigation.

In summary, we identify IL-22 as a critical antiviral effector cytokine in peripheral sites of MCMV infection and identified the recruitment of neutrophils as a mechanism through which IL-22 affords antiviral protection. These data define neutrophils as an important antiviral effector cell population that acts during the initial stages of CMV infection and uncover the TRAIL/TRAILR pathway as a mechanism through which neutrophils exert antiviral control.

EXPERIMENTAL PROCEDURES

Mice, Viral Infections, and Treatments

All experiments were conducted under a UK Home Office project license (PPL 30/2442 and 30/2969). WT C57BL/6 mice were purchased (Harlan), *RAG1*$^{-/-}$ mice were bred in-house, *IL-22*$^{-/-}$ mice were kindly provided by Jean-Christophe Renauld (Ludwig Institute, Brussels), and IL-22RA1 reporter mice were generated as described in the Supplemental Experimental Procedures. MCMV Smith strain (ATCC) was prepared in BALB/c salivary glands and purified over a sorbital gradient. Virus from homogenized organs and tissue culture supernatants were titered on 3T3 cells. Mice were infected intraperitoneally (i.p.) with 3×10^4 pfu MCMV and weighed daily. Some mice were injected i.p. with 200 μg αNK1.1 (clone PK136, BioXCell) or IgG control on days −2, 0, and +2 p.i. For T cell depletion, mice were injected (i.p.) with 200 μg αCD4 antibody (100 μg clone YTS191, 100 μg clone YTS3) and 200 μg αCD8 antibody (100 μg clone YTS156, 100 μg clone YTS169, all in-house). Neutrophils were depleted with 100 μg αLy6G (clone 1A8, BioXCell); for TRAIL neutralization mice were administered 250 μg of αTRAIL (clone N2B2, Biolegend), and for IL-22 neutralization mice were administered i.v. 50 μg goat IgG (Chemicon) or αIL-22 (R&D Systems). Some mice were treated with 100 μg IgG or αCXCL1 (clone 124014, R&D Systems). All administrations were day 0 and, for 4-day experiments, 2 p.i.

Flow Cytometry

Leukocytes were isolated from murine tissue as previously described (Stacey et al., 2011) and stained with Live/Dead Fixable Aqua (Invitrogen), incubated with Fc block (eBioscience), and stained for surface markers with a combination of antibodies listed in the Supplemental Experimental Procedures. To detect IL-22-secreting cells, cells were incubated in brefeldin A (Sigma-Aldrich) ± IL-23 (50 μg/ml, R&D Systems) ± 50 ng/ml PMA and 500 ng/ml ionomycin (both Sigma-Aldrich) prior to surface staining and permeabilization with saponin buffer, stained with αIL-22, αIFN-γ, αgranzyme B, and/or αRORγT. To measure CD11b expression by neutrophils, tissues remained on ice during processing and red blood cells were not lysed, to avoid cell stimulation prior to staining. For TRAILR expression analysis, fibroblasts were infected with MCMV at a moi of 0.2 and stained with αTRAILR (clone 5D5-1, eBioscience). Further details are provided in the Supplemental Experimental Procedures.

Cytokine/Chemokine Protein Measurement

Excized lungs, livers, and spleens (~50 mg) were weighed, washed in PBS, and homogenized in DMEM. Supernatants were assayed for cytokines by cytometric bead array (eBioscience) or, for IL-22 and CXCL1, ELISA (eBioscience and R&D Systems, respectively). In some experiments, 3T3 fibroblasts and SGC1 cells (determined by FACS to be IL-22R$^-$ and IL-22R$^+$, respectively, data not shown) were infected with MCMV (moi 0.5) or treated with 50 ng/ml IL-22 (R&D Systems) for 30 min prior to washing. CXCL1 protein was assayed by ELISA after 6 hr.

Histological Analysis

To identify IL-22RA$^+$ cells, tissue sections from WT and *IL-22ra1*-LacZ mice were stained overnight in 0.1% (w/v) 5-bromo-4-chloro-3-indolyl-beta-D-galactosidase (X-gal; Invitrogen), and reporter activity identified under light microscopy (Zeiss Axiovert 200M) with AxioVision version 4.8.2 software. Neutrophil colocalization was assessed in paraffin-embedded liver sections stained with αm123 and αLy6G and visualized with DAB (Vector Labs) and VIP Chromogen solution (Vector Labs), respectively. Further details are in the Supplemental Experimental Procedures.

Assessment of Gene Expression

RNA was extracted from tissue by RNA easy kit (QIAGEN), cDNA synthesized with a TaqMan reverse transcription kit (Applied Biosystems), and gene expression determined by quantitative PCR using a Mini Opticon (Bio-Rad) and Platinum SYBR green mastermix reagent (Biorad), using primers listed in Table S1.

Assessment of Neutrophil Antiviral Activity

Splenic neutrophils were purified from $RAG^{-/-}$ mice using a negative selection kit (Stemcell technologies) and a positive selection kit (Miltenyi Biotec) as described in the Supplemental Experimental Procedures, and incubated with MCMV-infected 3T3s (moi 0.02). MCMV in supernatants was measured 7 days later by plaque assay. Freeze-thawed neutrophils were nonviable controls. Some wells received inhibitory reagents: αTRAIL (10 μg/ml, clone N2B2, Biolegend), αFasL (10 μg/ml, clone 101626, R&D Systems), αIFNα (10 μg/ml, clone RMMA-1, PBL Interferon Source), αIFNβ (10 μg/ml, clone RMMB-1, PBL Interferon Source), αIFNγ (10 μg/ml, clone XMG1.2, in house), αTNF-α (10 μg/ml, XT22, in house), and iNOS inhibitors (10 μM, 1400W, Sigma-Aldrich). Some MCMV-infected fibroblasts were treated with TRAIL or FAS (R&D Systems) at concentrations stated in Figure 7.

Statistics

Statistical significance was determined using the Mann-Whitney *U* test (viral load analysis) or the two-tailed Student's t test (weight loss, flow cytometry, ELISA); $^*p \leq 0.05$, $^{**}p \leq 0.01$, $^{***}p \leq 0.001$.

SUPPLEMENTAL INFORMATION

Supplemental Information includes four figures, one table, and Supplemental Experimental Procedures and can be found with this article at http://dx.doi.org/10.1016/j.chom.2014.03.003.

ACKNOWLEDGMENTS

We thank Peter Ghazal and Bernhard Moser for helpful discussion, Anwen Williams for tissue processing, Stipan Jonjić for the kind gifts of αm06 and αm123 antibodies, Jean-Christophe Renauld for kind provision of $IL\text{-}22^{-/-}$ mice, David Withers for technical advice, and Ann Ager for critical discussions. This work was funded by the Wellcome Trust Senior Research fellowship awarded to I.R.H. (WT098026MA); project grants from the MRC (G1000236), BBSRC (BBF0098361), and Wellcome Trust (WT090323MA) awarded to G.W.G.W.; a NIH grant (AI101423) awarded to C.A.B.; a Cardiff University/MRC studentship (awarded to M.A.S.); and a Cardiff University President's Scholarship studentship (awarded to G.S.).

Received: August 7, 2013
Revised: November 26, 2013
Accepted: March 4, 2014
Published: April 9, 2014

REFERENCES

Andoniou, C.E., van Dommelen, S.L., Voigt, V., Andrews, D.M., Brizard, G., Asselin-Paturel, C., Delale, T., Stacey, K.J., Trinchieri, G., and Degli-Esposti, M.A. (2005). Interaction between conventional dendritic cells and natural killer cells is integral to the activation of effective antiviral immunity. Nat. Immunol. *6*, 1011–1019.

Andrews, D.M., Scalzo, A.A., Yokoyama, W.M., Smyth, M.J., and Degli-Esposti, M.A. (2003). Functional interactions between dendritic cells and NK cells during viral infection. Nat. Immunol. *4*, 175–181.

Aujla, S.J., Chan, Y.R., Zheng, M., Fei, M., Askew, D.J., Pociask, D.A., Reinhart, T.A., McAllister, F., Edeal, J., Gaus, K., et al. (2008). IL-22 mediates mucosal host defense against Gram-negative bacterial pneumonia. Nat. Med. *14*, 275–281.

Bai, F., Kong, K.F., Dai, J., Qian, F., Zhang, L., Brown, C.R., Fikrig, E., and Montgomery, R.R. (2010). A paradoxical role for neutrophils in the pathogenesis of West Nile virus. J. Infect. Dis. *202*, 1804–1812.

Banks, T.A., Rickert, S., Benedict, C.A., Ma, L., Ko, M., Meier, J., Ha, W., Schneider, K., Granger, S.W., Turovskaya, O., et al. (2005). A lymphotoxin-IFN-beta axis essential for lymphocyte survival revealed during cytomegalovirus infection. J. Immunol. *174*, 7217–7225.

Billerbeck, E., Kang, Y.H., Walker, L., Lockstone, H., Grafmueller, S., Fleming, V., Flint, J., Willberg, C.B., Bengsch, B., Seigel, B., et al. (2010). Analysis of CD161 expression on human CD8+ T cells defines a distinct functional subset with tissue-homing properties. Proc. Natl. Acad. Sci. USA *107*, 3006–3011.

Biron, C.A., Byron, K.S., and Sullivan, J.L. (1989). Severe herpesvirus infections in an adolescent without natural killer cells. N. Engl. J. Med. *320*, 1731–1735.

Bukowski, J.F., Woda, B.A., and Welsh, R.M. (1984). Pathogenesis of murine cytomegalovirus infection in natural killer cell-depleted mice. J. Virol. *52*, 119–128.

Cohen, N.R., Brennan, P.J., Shay, T., Watts, G.F., Brigl, M., Kang, J., Brenner, M.B., and Consortium, I.P.; ImmGen Project Consortium (2013). Shared and distinct transcriptional programs underlie the hybrid nature of iNKT cells. Nat. Immunol. *14*, 90–99.

Diehl, G.E., Yue, H.H., Hsieh, K., Kuang, A.A., Ho, M., Morici, L.A., Lenz, L.L., Cado, D., Riley, L.W., and Winoto, A. (2004). TRAIL-R as a negative regulator of innate immune cell responses. Immunity *21*, 877–889.

French, A.R., Pingel, J.T., Wagner, M., Bubic, I., Yang, L., Kim, S., Koszinowski, U., Jonjic, S., and Yokoyama, W.M. (2004). Escape of mutant double-stranded DNA virus from innate immune control. Immunity *20*, 747–756.

Futosi, K., Fodor, S., and Mócsai, A. (2013). Neutrophil cell surface receptors and their intracellular signal transduction pathways. Int. Immunopharmacol. *17*, 638–650.

Guabiraba, R., Besnard, A.G., Marques, R.E., Maillet, I., Fagundes, C.T., Conceição, T.M., Rust, N.M., Charreau, S., Paris, I., Lecron, J.C., et al. (2013). IL-22 modulates IL-17A production and controls inflammation and tissue damage in experimental dengue infection. Eur. J. Immunol. *43*, 1529–1544.

Guo, H., and Topham, D.J. (2010). Interleukin-22 (IL-22) production by pulmonary Natural Killer cells and the potential role of IL-22 during primary influenza virus infection. J. Virol. *84*, 7750–7759.

Hayakawa, Y., and Smyth, M.J. (2006). CD27 dissects mature NK cells into two subsets with distinct responsiveness and migratory capacity. J. Immunol. *176*, 1517–1524.

Hennig, B.J., Frodsham, A.J., Hellier, S., Knapp, S., Yee, L.J., Wright, M., Zhang, L., Thomas, H.C., Thursz, M., and Hill, A.V. (2007). Influence of IL-10RA and IL-22 polymorphisms on outcome of hepatitis C virus infection. Liver Int. *27*, 1134–1143.

Hsu, K.M., Pratt, J.R., Akers, W.J., Achilefu, S.I., and Yokoyama, W.M. (2009). Murine cytomegalovirus displays selective infection of cells within hours after systemic administration. J. Gen. Virol. *90*, 33–43.

Jaeger, B.N., Donadieu, J., Cognet, C., Bernat, C., Ordoñez-Rueda, D., Barlogis, V., Mahlaoui, N., Fenis, A., Narni-Mancinelli, E., Beaupain, B., et al. (2012). Neutrophil depletion impairs natural killer cell maturation, function, and homeostasis. J. Exp. Med. *209*, 565–580.

Jang, J.E., Hyun, S.Y., Kim, Y.D., Yoon, S.H., Hwang, D.Y., Kim, S.J., Kim, Y., Kim, J.S., Cheong, J.W., and Min, Y.H. (2012). Risk factors for progression from cytomegalovirus viremia to cytomegalovirus disease after allogeneic hematopoietic stem cell transplantation. Biol. Blood Marrow Transplant. *18*, 881–886.

Jenne, C.N., Wong, C.H., Zemp, F.J., McDonald, B., Rahman, M.M., Forsyth, P.A., McFadden, G., and Kubes, P. (2013). Neutrophils recruited to sites of infection protect from virus challenge by releasing neutrophil extracellular traps. Cell Host Microbe *13*, 169–180.

Juno, J.A., Keynan, Y., and Fowke, K.R. (2012). Invariant NKT cells: regulation and function during viral infection. PLoS Pathog. *8*, e1002838.

Kang, Y.H., Seigel, B., Bengsch, B., Fleming, V.M., Billerbeck, E., Simmons, R., Walker, L., Willberg, C.B., Barnes, E.J., Bhagwanani, A., et al. (2012). CD161(+)CD4(+) T cells are enriched in the liver during chronic hepatitis and associated with co-secretion of IL-22 and IFN-γ. Front. Immunol. *3*, 346.

Kim, C.J., Nazli, A., Rojas, O.L., Chege, D., Alidina, Z., Huibner, S., Mujib, S., Benko, E., Kovacs, C., Shin, L.Y., et al. (2012). A role for mucosal IL-22 production and Th22 cells in HIV-associated mucosal immunopathogenesis. Mucosal Immunol. *5*, 670–680.

Koga, Y., Matsuzaki, A., Suminoe, A., Hattori, H., and Hara, T. (2004). Neutrophil-derived TNF-related apoptosis-inducing ligand (TRAIL): a novel mechanism of antitumor effect by neutrophils. Cancer Res. *64*, 1037–1043.

Kumar, P., Thakar, M.S., Ouyang, W., and Malarkannan, S. (2013). IL-22 from conventional NK cells is epithelial regenerative and inflammation protective during influenza infection. Mucosal Immunol. *6*, 69–82.

La Gruta, N.L., Kedzierska, K., Stambas, J., and Doherty, P.C. (2007). A question of self-preservation: immunopathology in influenza virus infection. Immunol. Cell Biol. *85*, 85–92.

Lee, H.S., Park, J.Y., Shin, S.H., Kim, S.B., Lee, J.S., Lee, A., Ye, B.J., and Kim, Y.S. (2012). Herpesviridae viral infections after chemotherapy without antiviral prophylaxis in patients with malignant lymphoma: incidence and risk factors. Am. J. Clin. Oncol. *35*, 146–150.

Milligan, G.N. (1999). Neutrophils aid in protection of the vaginal mucosae of immune mice against challenge with herpes simplex virus type 2. J. Virol. *73*, 6380–6386.

Missé, D., Yssel, H., Trabattoni, D., Oblet, C., Lo Caputo, S., Mazzotta, F., Pène, J., Gonzalez, J.P., Clerici, M., and Veas, F. (2007). IL-22 participates in an innate anti-HIV-1 host-resistance network through acute-phase protein induction. J. Immunol. *178*, 407–415.

Orange, J.S., and Biron, C.A. (1996). An absolute and restricted requirement for IL-12 in natural killer cell IFN-gamma production and antiviral defense. Studies of natural killer and T cell responses in contrasting viral infections. J. Immunol. *156*, 1138–1142.

Ouyang, W., Rutz, S., Crellin, N.K., Valdez, P.A., and Hymowitz, S.G. (2011). Regulation and functions of the IL-10 family of cytokines in inflammation and disease. Annu. Rev. Immunol. *29*, 71–109.

Pellegrini, M., Calzascia, T., Toe, J.G., Preston, S.P., Lin, A.E., Elford, A.R., Shahinian, A., Lang, P.A., Lang, K.S., Morre, M., et al. (2011). IL-7 engages multiple mechanisms to overcome chronic viral infection and limit organ pathology. Cell *144*, 601–613.

Penfold, M.E., Dairaghi, D.J., Duke, G.M., Saederup, N., Mocarski, E.S., Kemble, G.W., and Schall, T.J. (1999). Cytomegalovirus encodes a potent alpha chemokine. Proc. Natl. Acad. Sci. USA *96*, 9839–9844.

Perrone, L.A., Plowden, J.K., García-Sastre, A., Katz, J.M., and Tumpey, T.M. (2008). H5N1 and 1918 pandemic influenza virus infection results in early and excessive infiltration of macrophages and neutrophils in the lungs of mice. PLoS Pathog. *4*, e1000115.

Sacher, T., Mohr, C.A., Weyn, A., Schlichting, C., Koszinowski, U.H., and Ruzsics, Z. (2012). The role of cell types in cytomegalovirus infection in vivo. Eur. J. Cell Biol. *91*, 70–77.

Sadik, C.D., Kim, N.D., and Luster, A.D. (2011). Neutrophils cascading their way to inflammation. Trends Immunol. *32*, 452–460.

Saitoh, T., Komano, J., Saitoh, Y., Misawa, T., Takahama, M., Kozaki, T., Uehata, T., Iwasaki, H., Omori, H., Yamaoka, S., et al. (2012). Neutrophil extracellular traps mediate a host defense response to human immunodeficiency virus-1. Cell Host Microbe *12*, 109–116.

Salazar-Mather, T.P., Orange, J.S., and Biron, C.A. (1998). Early murine cytomegalovirus (MCMV) infection induces liver natural killer (NK) cell inflammation and protection through macrophage inflammatory protein 1alpha (MIP-1alpha)-dependent pathways. J. Exp. Med. *187*, 1–14.

Satoh-Takayama, N., Vosshenrich, C.A., Lesjean-Pottier, S., Sawa, S., Lochner, M., Rattis, F., Mention, J.J., Thiam, K., Cerf-Bensussan, N., Mandelboim, O., et al. (2008). Microbial flora drives interleukin 22 production in intestinal NKp46+ cells that provide innate mucosal immune defense. Immunity *29*, 958–970.

Schneider, K., Loewendorf, A., De Trez, C., Fulton, J., Rhode, A., Shumway, H., Ha, S., Patterson, G., Pfeffer, K., Nedospasov, S.A., et al. (2008). Lymphotoxin-mediated crosstalk between B cells and splenic stroma promotes the initial type I interferon response to cytomegalovirus. Cell Host Microbe *3*, 67–76.

Shellam, G., Redwood, A., Smith, L., and Gorman, S. (2006). Mouse cytomegalovirus and other herpesviruses. In The Mouse in Biomedical Research, J. Fox, S. Barthold, M. Davisson, C. Newcomer, and F. Quimby, eds. (New York: Academic Press), pp. 1–48.

Smalls-Mantey, A., Connors, M., and Sattentau, Q.J. (2013). Comparative efficiency of HIV-1-infected T cell killing by NK cells, monocytes and neutrophils. PLoS ONE *8*, e74858.

Smith, W., Tomasec, P., Aicheler, R., Loewendorf, A., Nemčovičová, I., Wang, E.C., Stanton, R.J., Macauley, M., Norris, P., Willen, L., et al. (2013). Human cytomegalovirus glycoprotein UL141 targets the TRAIL death receptors to thwart host innate antiviral defenses. Cell Host Microbe *13*, 324–335.

Sonnenberg, G.F., Fouser, L.A., and Artis, D. (2011). Border patrol: regulation of immunity, inflammation and tissue homeostasis at barrier surfaces by IL-22. Nat. Immunol. *12*, 383–390.

Stacey, M.A., Marsden, M., Wang, E.C.Y., Wilkinson, G.W.G., and Humphreys, I.R. (2011). IL-10 restricts activation-induced death of NK cells during acute murine cytomegalovirus infection. J. Immunol. *187*, 2944–2952.

Stoddart, C.A., Cardin, R.D., Boname, J.M., Manning, W.C., Abenes, G.B., and Mocarski, E.S. (1994). Peripheral blood mononuclear phagocytes mediate dissemination of murine cytomegalovirus. J. Virol. *68*, 6243–6253.

Tang, W., Wang, W., Zhang, Y., Liu, S., Liu, Y., and Zheng, D. (2009). TRAIL receptor mediates inflammatory cytokine release in an NF-kappaB-dependent manner. Cell Res. *19*, 758–767.

Tate, M.D., Deng, Y.M., Jones, J.E., Anderson, G.P., Brooks, A.G., and Reading, P.C. (2009). Neutrophils ameliorate lung injury and the development of severe disease during influenza infection. J. Immunol. *183*, 7441–7450.

Tate, M.D., Ioannidis, L.J., Croker, B., Brown, L.E., Brooks, A.G., and Reading, P.C. (2011). The role of neutrophils during mild and severe influenza virus infections of mice. PLoS ONE *6*, e17618.

Tecchio, C., Huber, V., Scapini, P., Calzetti, F., Margotto, D., Todeschini, G., Pilla, L., Martinelli, G., Pizzolo, G., Rivoltini, L., and Cassatella, M.A. (2004). IFNalpha-stimulated neutrophils and monocytes release a soluble form of TNF-related apoptosis-inducing ligand (TRAIL/Apo-2 ligand) displaying apoptotic activity on leukemic cells. Blood *103*, 3837–3844.

Tessmer, M.S., Reilly, E.C., and Brossay, L. (2011). Salivary gland NK cells are phenotypically and functionally unique. PLoS Pathog. *7*, e1001254.

Tsai, H.H., Frost, E., To, V., Robinson, S., Ffrench-Constant, C., Geertman, R., Ransohoff, R.M., and Miller, R.H. (2002). The chemokine receptor CXCR2 controls positioning of oligodendrocyte precursors in developing spinal cord by arresting their migration. Cell *110*, 373–383.

Tumpey, T.M., Chen, S.H., Oakes, J.E., and Lausch, R.N. (1996). Neutrophil-mediated suppression of virus replication after herpes simplex virus type 1 infection of the murine cornea. J. Virol. *70*, 898–904.

van Dommelen, S.L., Sumaria, N., Schreiber, R.D., Scalzo, A.A., Smyth, M.J., and Degli-Esposti, M.A. (2006). Perforin and granzymes have distinct roles in defensive immunity and immunopathology. Immunity *25*, 835–848.

Verma, S., Wang, Q., Chodaczek, G., and Benedict, C.A. (2013). Lymphoid-tissue stromal cells coordinate innate defense to cytomegalovirus. J. Virol. *87*, 6201–6210.

Wolk, K., Kunz, S., Witte, E., Friedrich, M., Asadullah, K., and Sabat, R. (2004). IL-22 increases the innate immunity of tissues. Immunity *21*, 241–254.

Zenewicz, L.A., Yancopoulos, G.D., Valenzuela, D.M., Murphy, A.J., Karow, M., and Flavell, R.A. (2007). Interleukin-22 but not interleukin-17 provides protection to hepatocytes during acute liver inflammation. Immunity *27*, 647–659.

Zhang, Y., Cobleigh, M.A., Lian, J.Q., Huang, C.X., Booth, C.J., Bai, X.F., and Robek, M.D. (2011). A proinflammatory role for interleukin-22 in the immune response to hepatitis B virus. Gastroenterology *141*, 1897–1906.

Zheng, Y., Valdez, P.A., Danilenko, D.M., Hu, Y., Sa, S.M., Gong, Q., Abbas, A.R., Modrusan, Z., Ghilardi, N., de Sauvage, F.J., and Ouyang, W. (2008). Interleukin-22 mediates early host defense against attaching and effacing bacterial pathogens. Nat. Med. *14*, 282–289.

Cell Host & Microbe, Volume *15*

Supplemental Information

Neutrophils Recruited by IL-22 in Peripheral Tissues Function as TRAIL-Dependent Antiviral Effectors against MCMV

Maria A. Stacey, Morgan Marsden, Tu Anh Pham N, Simon Clare, Garry Dolton, Gabrielle Stack, Emma Jones, Paul Klenerman, Awen M. Gallimore, Philip R. Taylor, Robert J. Snelgrove, Trevor D. Lawley, Gordon Dougan, Chris A. Benedict, Simon A. Jones, Gavin W.G. Wilkinson, and Ian R. Humphreys

Figure S1

Lung

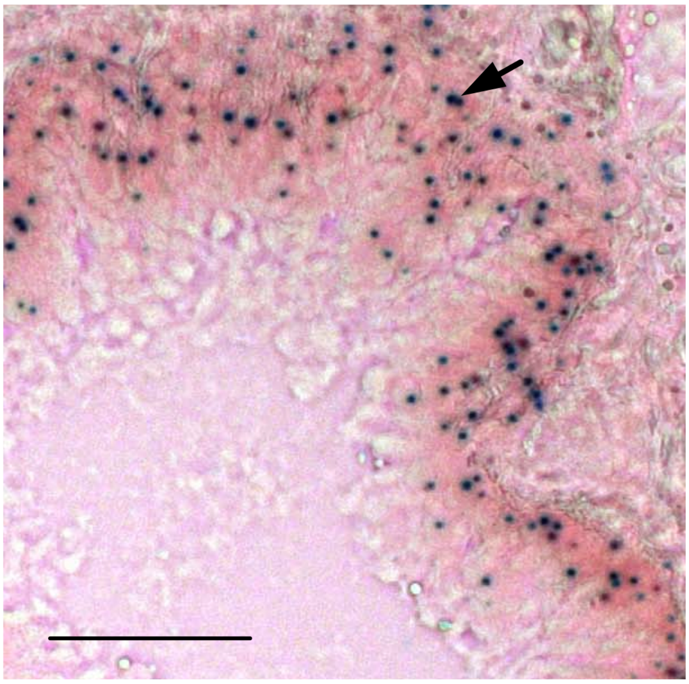

Liver

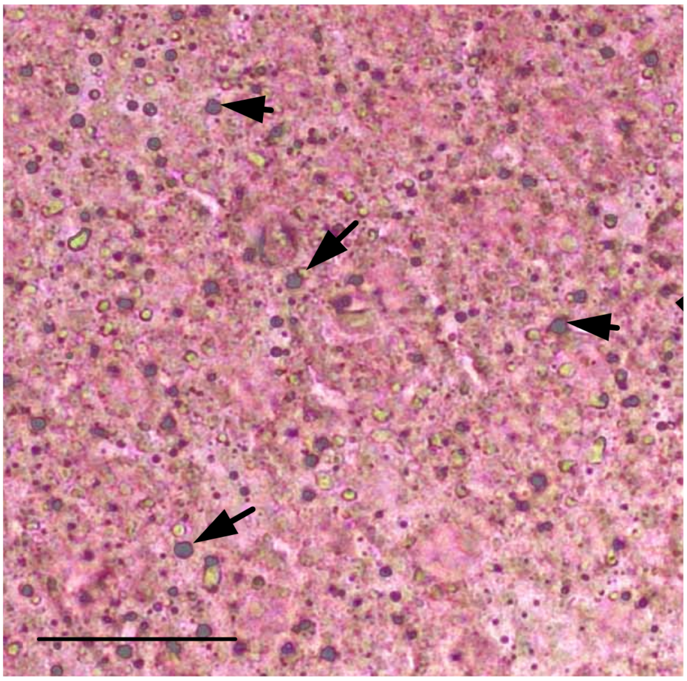

Figure S1, Related to Figure 1: IL-22R is expressed in the lung and liver. To assess *Il22ra1*-driven β-galactosidase reporter activity, lung (top) and liver (bottom) sections from *IL-22ra1*-LacZ reporter mice were incubated in X-gal. Arrows depict $LacZ^+$ epithelial cells (lung) and hepatocytes (liver). Bar = 20μM in all images.

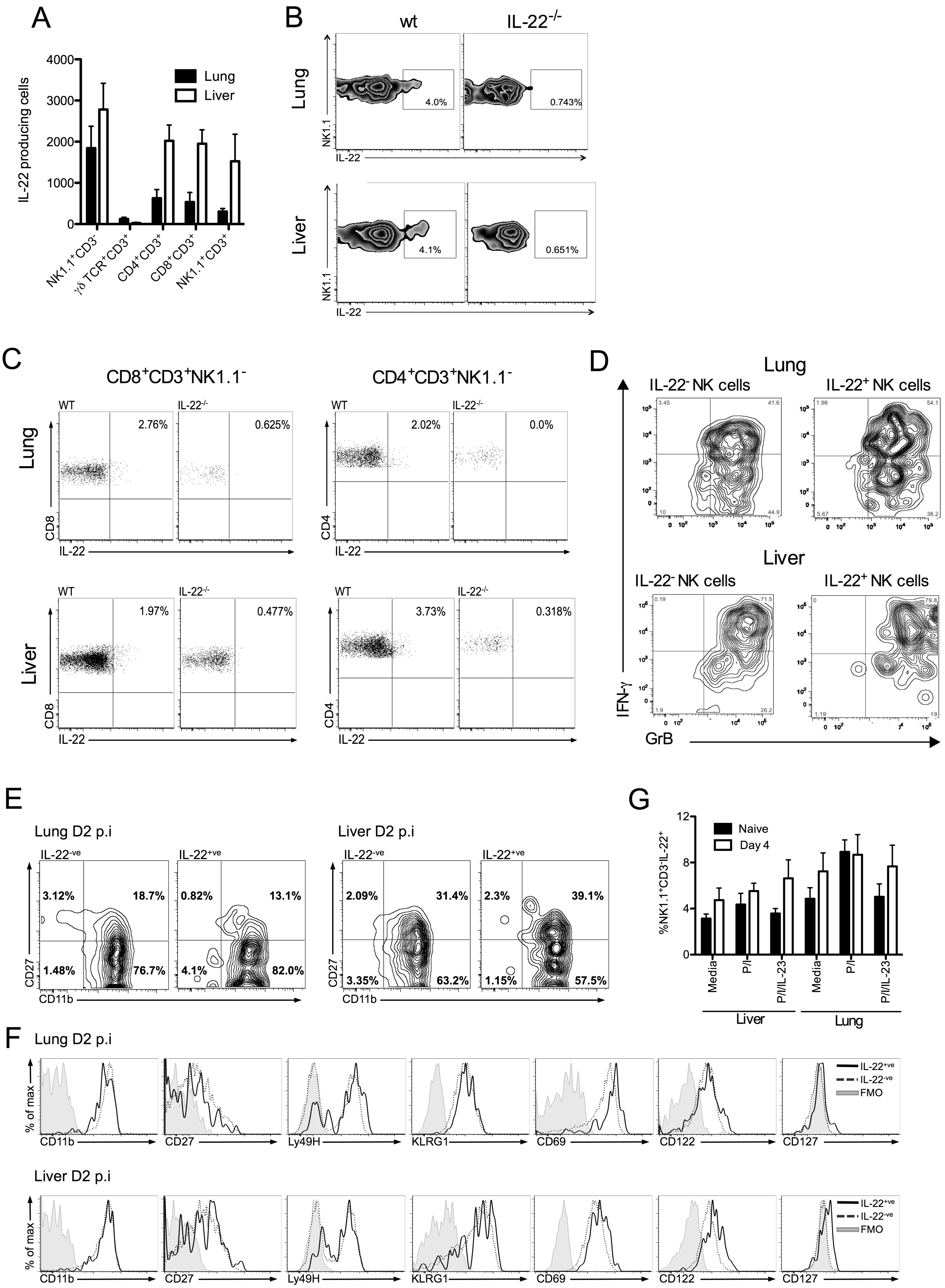
A
IL-22 producing cells
Lung
Liver
NK1.1+CD3-
γδ TCR+CD3+
CD4+CD3+
CD8+CD3+
NK1.1+CD3+
B
wt
IL-22-/-
Lung
Liver
NK1.1
IL-22
4.0%
0.743%
4.1%
0.651%
C
CD8+CD3+NK1.1-
CD4+CD3+NK1.1-
WT
IL-22-/-
2.76%
0.625%
2.02%
0.0%
1.97%
0.477%
3.73%
0.318%
D
Lung
Liver
IL-22- NK cells
IL-22+ NK cells
IFN-γ
GrB
E
Lung D2 p.i
Liver D2 p.i
IL-22-ve
IL-22+ve
CD27
CD11b
3.12%
18.7%
1.48%
76.7%
0.82%
13.1%
4.1%
82.0%
2.09%
31.4%
3.35%
63.2%
2.3%
39.1%
1.15%
57.5%
G
%NK1.1+CD3-IL-22+
Naive
Day 4
Media
P/I
P/I/IL-23
Liver
Lung
F
Lung D2 p.i
Liver D2 p.i
% of max
CD11b
CD27
Ly49H
KLRG1
CD69
CD122
CD127
IL-22+ve
IL-22-ve
FMO

Figure S2

Figure S2, Related to Figure 2. IL-22 is expressed by conventional NK cells and T cells in response to MCMV infection. (A) IL-22 expressing leukocytes in lung and liver tissue 2 days post-MCMV infection were quantified by flow cytometry. Results are shown as mean ± SEM of 5 mice. (B) Representative plots of IL-22 expression by $NK1.1^{+}CD3^{-}$ cells in wt and $IL\text{-}22^{-/-}$ mice d2 pi. (C) Representative FACS plots of IL-22 expression by wt and $IL\text{-}22^{-/-}$ $CD8^{+}CD3^{-}NK1.1^{-}$ (left) and $CD4^{+}CD3^{-}NK1.1^{-}$ (right) isolated from the lung (top) and liver (bottom) 4 days post-infection. (D) Representative plots of granzyme B and IFN–γ co-expression by $IL\text{-}22^{+}$ and $IL\text{-}22^{-}$ cells isolated from the lungs and livers 4 days pi. (E) Representative FACS plots of CD27 and CD11b co-expression by $IL\text{-}22^{+}$ and $IL\text{-}22^{-}$ NK cells in the lung and liver d2 pi and (F) representative histogram overlays of expression by $IL\text{-}22^{+}$ (solid line) and $IL\text{-}22^{-}$ (dotted line) of NK cell markers. Results represent 2 experiments. (G) IL-22 expression by $NK1.1^{+}CD3^{-}$ cells from naïve and MCMV-infected mice after 4 hours incubation in the presence of brefeldin A, PMA/ionomycin or PMA/ionomycin/IL-23. Mean ± SEM of 4 mice/group is shown. Data is representative of 2 independent experiments.

Figure S3

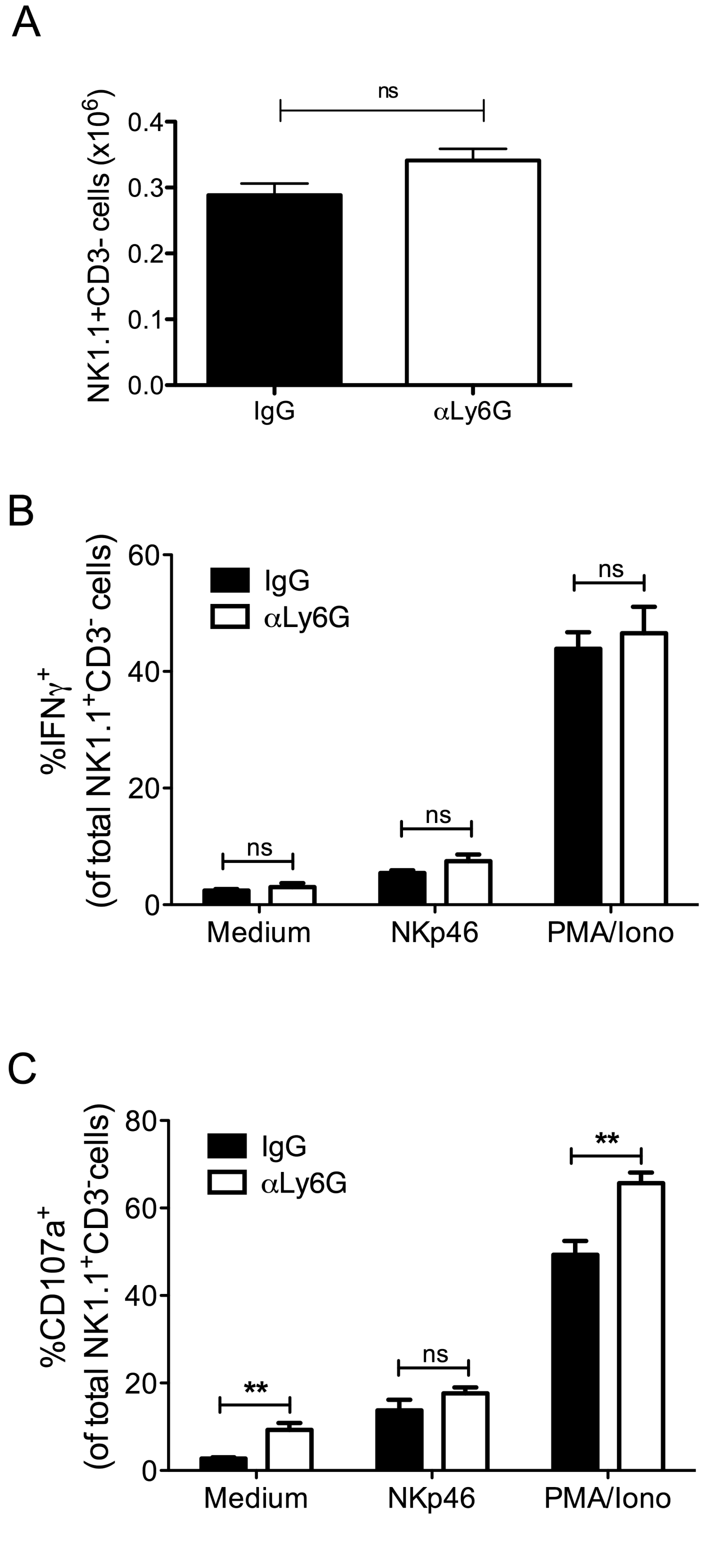
A
NK1.1+CD3- cells (x10^6)
0.0
0.1
0.2
0.3
0.4
ns
IgG
αLy6G
B
%IFNγ+ (of total NK1.1+CD3- cells)
0
20
40
60
IgG
αLy6G
ns
ns
ns
Medium
NKp46
PMA/Iono
C
%CD107a+ (of total NK1.1+CD3-cells)
0
20
40
60
80
IgG
αLy6G
**
ns
**
Medium
NKp46
PMA/Iono

Figure S3, Related to Figure 6. Neutrophil depletion during MCMV infection does not impair NK cell function. MCMV-infected mice were treated with IgG (closed bars) or αLy6G (open bars). (A) After 4 days, leukocytes were isolated from the liver and $NK1.1^{+}CD3^{-}$ cells were quantified. (B & C) CD107a mobilization (B) and IFN–γ (C) expression by $NK1.1^{+}CD3^{-}$ cells was measured following 4 hours of incubation in the presence of monensin (A) or brefeldin A (B) in media +/- plate-bound αNKp46 or PMA/ionomycin. Results are expressed as % positive of all $NK1.1^{+}$ $CD3^{-}$ cells, as assessed by flow cytometry. Mean + SEM in all graphs is shown from 6 mice per group. **p≤0.01, ns=not significant.

Figure S4

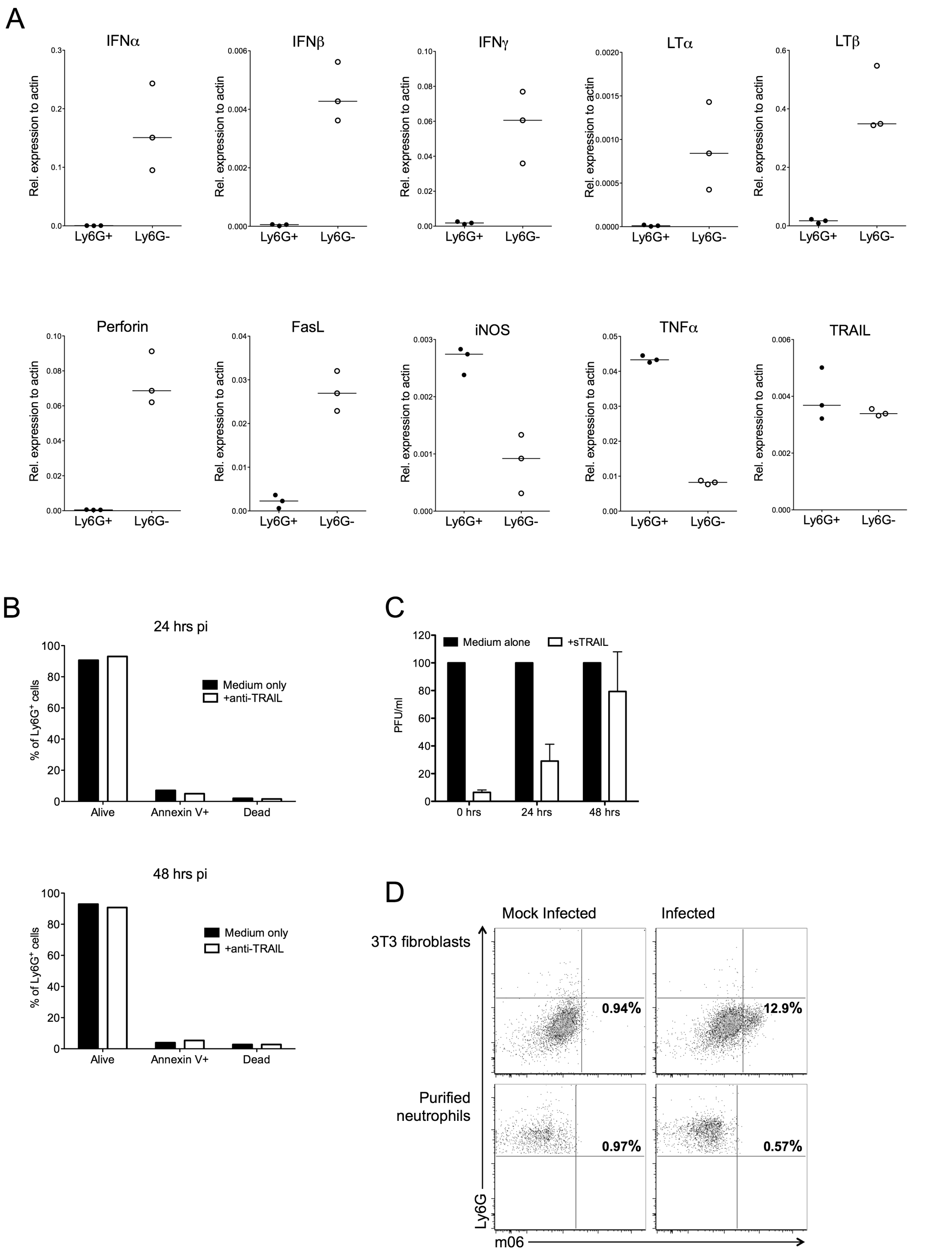
A
IFNα
IFNβ
IFNγ
LTα
LTβ
Perforin
FasL
iNOS
TNFα
TRAIL
Rel. expression to actin
Ly6G+
Ly6G-
B
24 hrs pi
48 hrs pi
% of Ly6G+ cells
Medium only
+anti-TRAIL
Alive
Annexin V+
Dead
C
Medium alone
+sTRAIL
PFU/ml
0 hrs
24 hrs
48 hrs
D
Mock Infected
Infected
3T3 fibroblasts
Purified neutrophils
0.94%
12.9%
0.97%
0.57%
Ly6G
m06

Figure S4, Related to Figure 7. Neutrophils limit MCMV replication via TRAIL and are not susceptible to MCMV infection. (A) Ly6G$^+$ and Ly6G$^-$ cells were purified from livers of MCMV-infected mice 2 days after infection and mRNA expression of antiviral effector genes assessed by qPCR. Individual mice + mean are shown and results represent 2 experiments. (B) Purified Ly6G$^+$ neutrophils were co-incubated with MCMV-infected 3T3 fibroblasts (MOI=0.02), and after 24 (top) and 48 (bottom) hours, neutrophil survival was assessed by the absence of Annexin V and live/dead aqua staining. Data represents 2 separate experiments. (C) Soluble TRAIL was added or not to MCMV-infected fibroblasts 0, 24 or 48 hours post-MCMV infection and virus production in supernatants were measured 7 days later. Data from 3 merged experiments are shown. (D) Purified Ly6G$^+$ neutrophils or 3T3 fibroblasts were mock-infected or infected with MCMV (MOI=0.2) and 24 hours later productively-infected cells were detected with αm06 antibody.

Supplementary Table 1, Related to Experimental Procedures. Primer sequences for qPCR analysis of gene expression.

Gene of interest	Forward primer	Reverse primer
β-actin	GGGCTATGCTCTCCCTCAC	GATGTCACGCACGATTTCC
IFNα	GTCAGTGTCAGAAGCTCCTGTGGC	CTATGGTCCAGGCACAGTGACTG
IFNβ	GAATCTCTCCTTTCTCCTG	CTGACAACCTCCCAGGCAC
IFNγ	GCCGTGGCAGTAACAGCC	AACGCTACACACTGCATCTGGG
LTα	AACCCAAGAATTGGATTCCAGG	TGTGACCCTTGAAACAACGGT
LTβ	TGTCTCCAGCTGCGGATTCTA	TTTGGCAGCTGTTGAACCC
Perforin	GAGAAGACCTATCAGGACCA	AGCCTGTGGTAAGCATG
FasL	TGAATTACCCATGTCCCCAG	AAACTGACCCTGGAGGAGCC
iNOS	GGCAGCCTGTGAGACCTTTG	GCATTGGAAGTGAAGCGTTTC
TNFα	CATCTTCTCAAAATTCGAGTGACAA	TGGGAGTAGACAAGGTACACCCC
TRAIL	CCTCTCGGAAAGGGCATTC	TCCTGCTCGATGACCAGCT
IL-22R	CTACGTGTGCCGAGTGAAGA	AAGCGTAGGGGTTGAAAGGT

Supplemental Experimental Procedures

IL-22R Reporter mice

The IL-22RA1 reporter mice were generated as part of the Sanger Mouse Genetics Project (MGP, www.knockoutmouse.org) using JM8 ES cells on a C57BL/6N background (Pettitt et al., 2009; Skarnes et al., 2011). The targeted *Il22ra1*$^{tm1a(KOMP)Wtsi}$ allele comprised a promoter-driven targeted DNA cassette encoding a neomycin-resistance marker, inserted into the first intron (http://www.sanger.ac.uk/mouseportal/search?query=il22ra1). The activity of the splice acceptor site within the neomycin cassette formed a truncated, non-functional mRNA containing only one of seven exons (ENSMUSE00000661894).

Flow cytometry

Leukocytes were stained for surface and intracellular markers with a combination of the following antibodies (from Biolegend, BD Biosciences, eBioscience, R and D Systems or Serotec): Ly6G (1A8), CD11b (M1/70), Ly6B (7/4), F4/80 (BM8), NK1.1 (PK136), CD3 (145-2C11), TCRγδ (GL3), Gr1 (RB6-8C5), CD4 (RM4-5), CD8 (53-6.7), TRAIL (N2B2), Ly49H (3D10), KLRG-1 (2F1), CD122 (TM-β1), CD27 (LG.3A10), CD127 (A7R34), CD69 (H1.2F3), IL-22 (IC582P) αIFN–γ (XMG1.2) and granzyme B (clone GB11). In some experiments, cells were also stained with αRORγT (clone B2D, eBioscience) according to published methodology (Sanos and Diefenbach, 2010). MCMV infection of neutrophils and 3T3 cells was assessed by intracellular staining with αm06 conjugated to APC (Innova biosciences).

All data were acquired on a BD FACS Canto II. Electronic compensation was performed with antibody-capture beads (BD Biosciences) stained separately with individual antibodies used in the experimental panel. A minimum of 30,000 events was acquired in each case and data analyzed using FlowJo software version 8.5.3 (TreeStar Inc.). Total numbers of different cell populations were calculated by multiplying the total number of viable leukocytes (assessed by trypan blue exclusion) by percent positive cells, as detected by flow cytometry.

Histological analysis of neutrophil and MCMV co-localization

To identify MCMV and neutrophil co-localization, paraffin-embedded liver sections were rehydrated after deparaffinising. Sections were then incubated with pre-warmed 0.01% trypsin for 15mins at 37°C. After blocking steps, sections were stained overnight 4°C with 2μg/ml αm123 (Croma 101, a kind gift from Stipan Jonjić, University of Rijeka) and visualized with DAB (Vector Labs). Slides were then counterstained with 2μg/ml αLy6G (1A8, Bioxcell) for 1 hour at room temperature, visualized with VIP Chromogen solution (Vector Labs) and counterstained with hematoxylin. Images were acquired using a Nikon Eclipse 80I microscope and Nikon ACT1 software.

Assessment of Ly6G^{+} and Ly6G^{-} gene expression

To examine neutrophil-specific gene expression, leukocytes were isolated from the livers of MCMV-infected mice, stained at 4°C with αLy6G-PE and separated by positive selection with αPE beads according to manufacturers

instructions (Miltenyi Biotec). RNA from positive (>80% Ly6G$^+$) and negative fractions (<2% Ly6G$^+$) was then extracted using an RNAeasy plus kit (Qiagen), treated with DNAse and cDNA was synthesized with a TaqMan reverse transcription kit (Applied Biosystems). Tissue RNA was extracted with RNAeasy kit and cDNA was synthesized as above. Gene expression was then assayed quantitative PCR using a Mini Opticon (Bio-Rad) and Platinum SYBR green mastermix reagent (Biorad). All primer sequences for gene detection are provided in Supplemental Table 1.

Neutrophil purification for *in vitro* killing assay

To purify neutrophils from RAG$^{-/-}$ spleens, splenocytes were first incubated with EasySep mouse neutrophil enrichment negative selection cocktail, washed and incubated with EasySep biotin selection cocktail. The cells were then incubated with EasySep magnetic particles and run through an LS column (Miltenyi). The negative fraction was then subjected to αLy6G-PE staining, and positive selection as described above. The purity of the separated cell fractions were assessed by flow cytometry to be ≥97%. In some experiments, neutrophils were isolated on days stated in legends and neutrophil death/apoptosis assessed with Live/Dead Fixable Aqua (Invitrogen) and Annexin-V (BD Biosciences), according to manufacturer's instructions.

Supplemental References

Pettitt, S.J., Liang, Q., Rairdan, X.Y., Moran, J.L., Prosser, H.M., Beier, D.R., Lloyd, K.C., Bradley, A., and Skarnes, W.C. (2009). Agouti C57BL/6N embryonic stem cells for mouse genetic resources. Nat Methods *6*, 493-495.

Sanos, S.L., and Diefenbach, A. (2010). Isolation of NK cells and NK-like cells from the intestinal lamina propria. Methods Mol Biol *612*, 505-517.

Skarnes, W.C., Rosen, B., West, A.P., Koutsourakis, M., Bushell, W., Iyer, V., Mujica, A.O., Thomas, M., Harrow, J., Cox, T., *et al.* (2011). A conditional knockout resource for the genome-wide study of mouse gene function. Nature *474*, 337-342.
